# The translocated virulence protein VirD5 causes DNA damage and mutation during *Agrobacterium*-mediated transformation of yeast

**DOI:** 10.1126/sciadv.add3912

**Published:** 2022-11-16

**Authors:** Xiaorong Zhang, Marjolein J. G. Hooykaas, G. Paul van Heusden, Paul J. J. Hooykaas

**Affiliations:** Institute of Biology, Leiden University, Leiden, Netherlands.

## Abstract

The soil bacterium *Agrobacterium tumefaciens* is a preferred gene vector not only for plants but also for fungi. *Agrobacterium* delivers a small set of virulence proteins into host cells concomitantly with transferred DNA (T-DNA) to support the transformation process. Here, we find that expression of one of these proteins, called VirD5, in yeast host cells causes replication stress and DNA damage. This can result in both genomic rearrangements and local mutations, especially small deletions. Delivery of VirD5 during cocultivation with *Agrobacterium* led to mutations in the yeast genome that were unlinked to the integration of T-DNA. This load of mutations can be prevented by using a *virD5* mutant for genome engineering, but this leads to a lower transformation frequency.

## Teaser

The natural genetic engineer *Agrobacterium tumefaciens* causes replication stress, DNA damage, and mutation in yeast cells.

## INTRODUCTION

In nature, *Agrobacterium tumefaciens* transforms plant cells at wound sites, resulting in tumor formation. The neoplastic properties of the plant cells in these tumors, called crown galls, are due to the presence of a segment of DNA, the transferred DNA (T-DNA), derived from the tumor-inducing (Ti) plasmid in the bacterium (*1*). The process of *Agrobacterium*-mediated transformation (AMT) has been described in several detailed reviews (*2*–*5*). Because of its natural ability to transform plant cells, *Agrobacterium* was engineered to become the preferred vector for the genetic modification of plants (*6*). Later, it was shown that *Agrobacterium* could also transform yeasts and fungi (*7*, *8*), and AMT has become the “silver bullet” for functional genomics of fungi (*9*). T-DNA integrates by nonhomologous recombination at a random position into the genome, thus causing insertion mutations (*10*). Libraries of T-DNA integration lines have therefore been used to select plants and fungi with specific phenotypes to be able to find genes (marked by T-DNA) linked to these phenotypes (*11*, *12*). However, it was found that, in T-DNA–transformed lines, mutations responsible for the phenotypes may be unlinked to the integrated T-DNA (*13*–*16*). These mutations were largely attributed to somaclonal variation as a consequence of plant cell and tissue culture (*15*, *16*). However, also after floral dip transformation, which does not include tissue culture steps, mutations have been observed that were unlinked to T-DNA (*14*, *15*). In a small pool of five transgenic lines analyzed by next-generation sequencing (NGS), the mutation frequency was within the range of spontaneous mutations occurring during seed propagation (*14*). Therefore, it is still questionable whether *Agrobacterium* is genotoxic like some other bacteria such as *Helicobacter pylori*, which delivers the virulence protein CagA into host cells to elicit BRCAness and genome instability (*17*).

Here, we have studied the *Agrobacterium* virulence protein VirD5, a large protein of 833 amino acids (*18*) that is translocated into host cells along with some other virulence proteins to support transformation (*19*–*21*). Expression of VirD5 in plant, yeast, and mammalian cells is toxic (*22*, *23*). VirD5 has two putative nuclear localization sequences (NLSs) that allow it to localize to the nucleus in host cells (*18*, *24*), where it interacts with several host proteins (*25*, *26*) including the transcription elongation factor Spt4 (*23*). It forms foci at the centromeres/kinetochores of the chromosomes (*23*) together with Spt4, which is enriched here (*27*). Via its N-terminal part, which includes a repetitive structure (*28*), VirD5 induces hyperactivation of the Aurora kinase Ipl1, causing chromosome missegregation during mitosis, leading to chromosome loss and aneuploidy (*23*, *28*). However, the N-terminal part of VirD5 (VirD5NT) is only weakly toxic to host cells in contrast to complete VirD5. Using yeast as a model, we describe here that expression of the C-terminal part of VirD5 (VirD5CT) or full VirD5 causes replication stress, DNA damage, and mutation in host cells. Our results show that mutations that are unlinked to T-DNA integration can be attributed at least in part to the action of VirD5. Mutations accompanying T-DNA transformation can be diminished by using an *Agrobacterium virD5* mutant instead of the wild-type helper strain for genome engineering, although this may lead to a lower transformation frequency.

## RESULTS

### Expression of VirD5CT inhibits growth and causes DNA damage

**Fig. 1.**
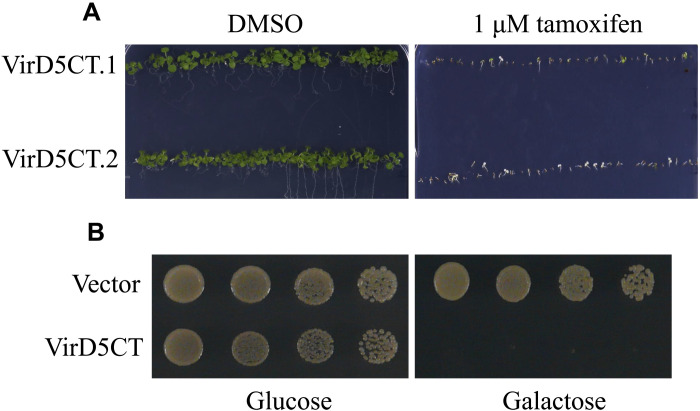
VirD5CT inhibits the growth of plant and yeast. (**A**) Two independent transgenic *A. thaliana* lines (VirD5CT.1 and VirD5CT.2) containing *virD5CT* driven by a tamoxifen-inducible promoter were grown on MS medium containing tamoxifen and without tamoxifen [dimethyl sulfoxide (DMSO)], respectively. (**B**) Yeast cells (BY4743) transformed with a high-copy number (pMVHis-VirD5CT) harboring *virD5CT* under the control of the *GAL1* promoter. Transformants were fivefold serially diluted and spotted onto MY medium containing either 2% glucose or 2% galactose and incubated for 3 days at 30°C.

Previously, we found that the expression of *virD5* inhibits the growth of plant, mammalian, and yeast cells (*22*, *23*). The VirD5NT (amino acids 1 to 505) causes chromosomal missegregation but is much less toxic than the full-length VirD5 protein (*23*, *28*). This suggested that the VirD5CT may be largely responsible for the toxicity of VirD5. We tested this by expressing VirD5CT, which encodes the C-terminal part (521 to 833 amino acids) of VirD5 under the control of an inducible promoter in yeast and *Arabidopsis thaliana*. As can be seen in Fig. 1, the expression of VirD5CT led to strong growth inhibition in both *A. thaliana* (Fig. 1A) and yeast (Fig. 1B).

**Fig. 2.**
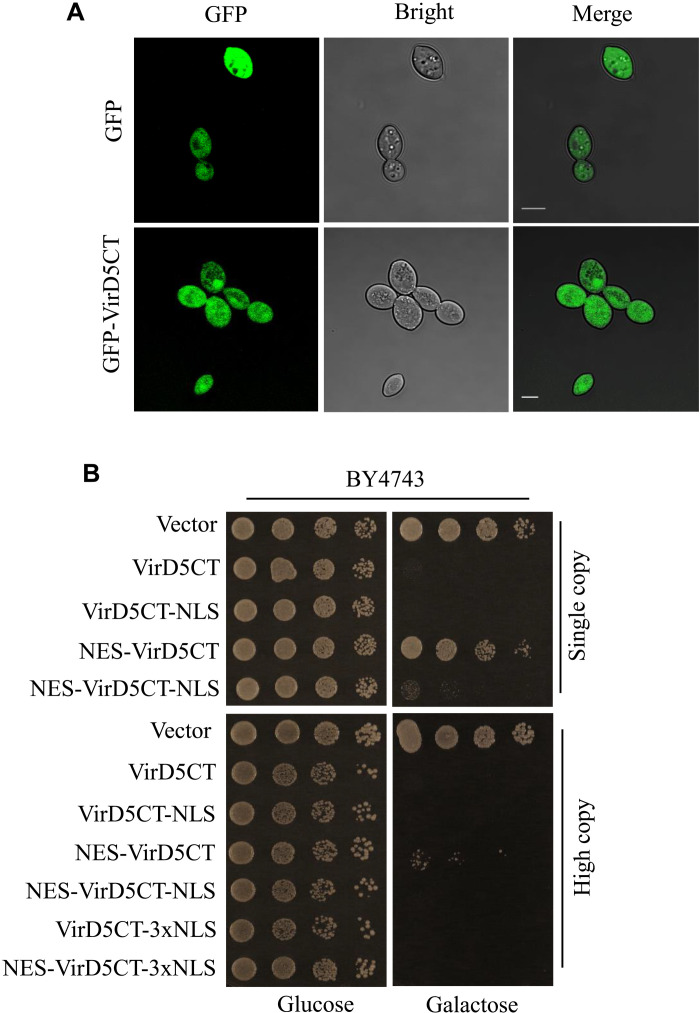
VirD5 localizes throughout the yeast cell but acts in the nucleus. (**A**) GFP-VirD5CT localizes throughout the yeast cell like GFP. (**B**) Toxicity of VirD5CT for yeast is dependent on the expression level (from a single-copy or multicopy plasmid) and its localization. Addition of a nuclear export sequence (NES) diminishes toxicity, suggesting that VirD5CT acts in the nucleus. Scale bars, 5 micrometer.

**Fig. 3.**
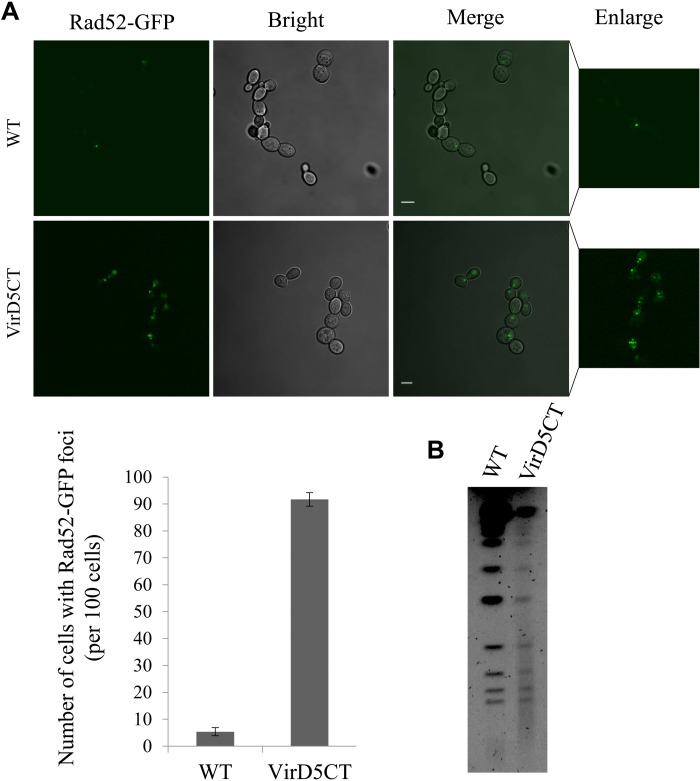
Expression of VirD5CT leads to genomic DNA damage. (**A**) Yeast cells expressing VirD5CT almost invariably show one or more Rad52-GFP foci indicative of DNA damage and repair in contrast to the isogenic yeast cells lacking VirD5CT, in which only, occasionally, a single focus appears, and quantification of the data below. (**B**) Visualization of the yeast chromosomes after pulse-field gel electrophoresis (PFGE) from yeast cells expressing VirD5CT in comparison with cells lacking VirD5CT. WT, wild type. Scale bars, 5 micrometer.

VirD5CT [labeled with green fluorescent protein (GFP)] is present throughout the yeast cell (Fig. 2A) in contrast to the full-length protein, which has two putative NLSs from amino acids 321 to 341 and from 766 to 783 (*18*), and is localized mainly to the nucleus (*23*, *24*). Addition of the nuclear export signal (NES) peptide from HIV diminished the toxicity of VirD5CT and allowed yeast colony formation, when expressed from a low–copy number plasmid (Fig. 2B). Addition of the NLS peptide from the simian virus 40 (SV40) at the C terminus of VirD5CT did not result in a visible change of toxicity. To exclude that addition of the NES at the N terminus of VirD5CT had led to an inactive protein, we added the SV40 NLS at the C terminus of NES-VirD5CT, resulting in NES-VirD5CT-NLS. This increased toxicity for yeast, with only some residual growth seen when expressed from the low–copy number vector, indicating that addition of the NES had not inactivated VirD5CT. As this indicated that VirD5CT like full VirD5 acted in the nucleus, we tested whether VirD5CT might cause DNA damage. To this end, we expressed VirD5CT in a yeast strain in which the genomic copy of the *RAD52* gene was tagged with GFP at its C terminus. The foci of Rad52, the key protein involved in homologous DNA repair, correspond to sites where genomic DNA is being repaired (*29*). As can be seen in Fig. 3A, more than 90% of the yeast cells expressing VirD5CT displayed green dots representing DNA repair foci in the nucleus, while only few cells lacking VirD5CT (1 to 5%) showed a single DNA repair focus. After cell cycle arrest in G_1_ in medium containing α-factor (5 μg/ml), we saw that numbers of Rad52-GFP foci started to increase after 40 min of regrowth, suggesting that VirD5CT causes DNA damage when cells are in the DNA replication phase (fig. S1).

As this suggested that VirD5CT caused DNA damage, we performed clamped homogeneous electrical field (CHEF) technology–based pulse-field gel electrophoresis (PFGE) to visualize the chromosomes of yeast after growth in the presence and absence of VirD5CT. As can be seen in Fig. 3B, bands representing the different yeast chromosomes can be seen in the lane with the genomic DNA from yeast cells not expressing VirD5CT, but only much fainter bands against the background of a smear were seen in the lane with genomic DNA from yeast expressing VirD5CT. This resembles the fragmentation of chromosomes that is observed after PFGE of chromosomal DNA isolated from yeast cells that have been treated with a DNA-damaging agent such as bleomycin or hydrogen peroxide (*30*). This suggests that the expression of VirD5CT had similarly caused chromosome breakage and degradation. The full VirD5 protein caused similar chromosome fragmentation as VirD5CT, but VirD5NT, which is responsible for chromosome missegregation, had no visible effect (fig. S2).

### VirD5CT interferes with DNA replication, and toxicity can be overcome by overexpression of subunits of the polymerase α–primase complex

**Fig. 4.**
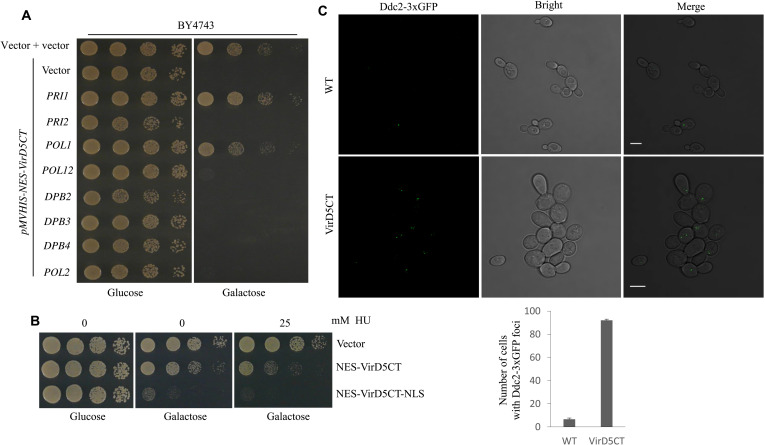
Expression of VirD5CT causes replication stress in yeast. (**A**) The growth inhibitory effect of NES-VirD5CT is diminished by overexpression of the catalytic subunits of primase–polymerase α. (**B**) The presence of a low dose of NES-VirD5CT or NES-VirD5CT-NLS makes yeast more sensitive to inhibition by hydroxyurea (HU). (**C**) Ddc2-GFP foci, indicative of replication stress, appear in yeast cells expressing VirD5CT. Quantification of the data in the graph below. Scale bars, 5 micrometer.

In hope to find the genes encoding the targets affected by VirD5CT, we screened the about 1500 clones of a yeast genomic tiling overexpression library, in which about 10-kb large genomic DNA fragments were present on a multicopy plasmid (*31*), for clones that could suppress the toxicity of VirD5CT. We found in this library two individual plasmids that suppressed the toxicity of VirD5CT. The first of these plasmids contained the genes *INP52*, *YNL105W*, *LEU4*, *YNL103W-A*, *MET4*, *POL1*, and *AVT4*, while the second contained *DJP1*, *IST3*, *PAN1*, *tE(UUC)I*, *YIR007W*, *PRI1*, *MSL1*, and *DSN1*. It was notable that each of these clones contained a gene encoding a subunit of the yeast DNA polymerase α–primase complex, *PRI1* and *POL1*, respectively. This complex plays an essential role in lagging strand DNA synthesis during replication and comprises four subunits, Pol1, Pol12, Pri1, and Pri2 (*32*). Subsequently, we retested several other clones from the library with replication-related genes, but only the two clones initially identified showed suppression. To determine whether overexpression of *PRI1* and *POL1* could suppress the toxicity of VirD5CT, the *PRI1* and *POL1* genes with their own promoters and terminators were separately cloned into high-copy number pRS425 and expressed in yeast cells expressing VirD5CT (Fig. 4A). In agreement with the results from the library screen, cells overexpressing either *PRI1* or *POL1* separately suppressed the toxicity of VirD5CT. However, clones containing genes for either of the noncatalytic subunits *PRI2* or *POL12* in the same pRS425 vector gave no noticeable suppression (Fig. 4A).

As overexpression of DNA polymerase α–primase rescued yeast from toxicity by VirD5, we assumed that VirD5 could interfere with DNA replication. If this would be the case, then replication in the presence of VirD5CT would probably be more sensitive to further disturbance by compounds such as hydroxyurea (HU). Therefore, we checked whether cells expressing VirD5CT are hypersensitive to HU. HU depletes the cell of deoxynucleotide triphosphates and thus leads to stalling of replication forks, which may eventually result in fork collapse and the formation of double-strand breaks (*33*). We used yeast cells expressing NES-VirD5CT or NES-VirD5CT-NLS from a single-copy vector for this experiment to be able to see the effects of a low dose of nuclear VirD5CT in this assay. As can be seen in Fig. 4B, control cells transformed with the empty vector (pRS315) showed no obvious growth inhibition in the presence of a low dose of HU. However, cells expressing NES-VirD5CT already showed slightly reduced growth in the presence of the drug, while growth was completely inhibited in yeast cells expressing NES-VirD5CT-NLS in the presence of the drug, indicating an increased susceptibility to HU and replication stress (Fig. 4B).

That DNA damage inflicted by VirD5CT arises in S phase suggests that VirD5CT either directly interferes with a component of the DNA replication machinery or causes DNA damage that leads to stalling of the replication fork. Such disturbances often give rise to the presence of segments of unreplicated single-stranded DNA (ssDNA) in the genome, which are bound by Replication protein A (RPA) that, in turn, recruits the Mec1-Ddc2 (ATR-ATRIP) complex that activates the replication checkpoint (*34*). We expressed Ddc2 labeled with 3xGFP in wild-type cells and in cells expressing VirD5CT to find out whether VirD5CT induces replication stress. As can be seen in Fig. 4C, while only 5% of the wild-type cells displayed Ddc2 foci, more than 90% of the cells expressing VirD5CT showed Ddc2 foci. This indicated that the presence of VirD5CT leads to frequent replication stress and activation of the replication checkpoint.

Stalled replication forks can be rescued in various ways, which may involve fork reversal to prevent fork collapse and subsequent fork repair that may entail translesion synthesis and homologous recombination by template switching to fill the remaining ssDNA gaps (*35*). We tested a set of DNA repair mutants for sensitivity to VirD5CT and found that mutants in *rad51* and *rad52* were hypersensitive to VirD5CT (figs. S3 and S4). These genes are very important not only for double-strand break repair by homologous recombination but also for replication fork protection (*36*). Besides, we found that mutations in genes coding for the Mre11-Rad50-Xrs2 complex, the Srs2 helicase, and the checkpoint protein Rad9 with similarity to mammalian 53BP1 were hypersensitive to VirD5CT. Otherwise, mutations in the genes for the 9-1-1 checkpoint proteins, the genes for the Rrm3 and Sgs1 helicases, or the Ku70 and Ku80 proteins involved in nonhomologous recombination behaved like the wild type when grown in the presence of VirD5CT (fig. S3). A mutation in the gene for the conserved transcription elongation factor Spt4 rescued growth in the presence of VirD5CT (fig. S4) like this rescued growth in the presence of VirD5 (*23*). Because VirD5 binds directly to the Spt4 protein in vivo, this suggests that VirD5-induced replication stress may be indirectly caused by the binding of VirD5 to transcription complexes, leading to transcription-replication conflicts.

### Expression of VirD5CT causes mutation

**Fig. 5.**
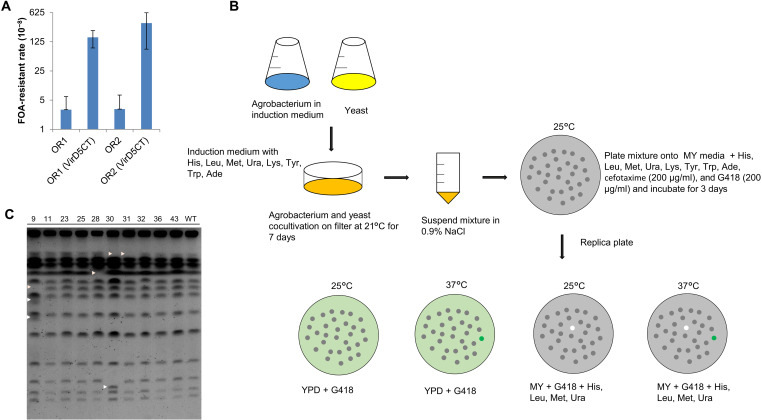
Expression of VirD5CT causes mutation. (**A**) An increased number of *URA3* mutations found in yeast cells expressing VirD5CT. Mutants were selected by their resistance to fluoroorotate (5-FOA). The *URA3* gene was located in chromosome III adjacent to *ARS306* in either of two orientations (OR1, strain YTAK001; and OR2, strain YTAK002). (**B**) Schematic overview of the protocol used to test whether translocated VirD5 is mutagenic in yeast. Yeast were transformed by the *Agrobacterium virD5* mutant and the isogenic wild-type strain containing binary vector pSDM8001 with the yeast-selectable KanMX marker. Transformants were selected for G418 resistance. As the KanMX maker in pSDM8001 is surrounded by homologous flanks from the yeast *PDA1* locus, the T-DNA integrates by homologous recombination at the *PDA1* locus in chromosome V. In populations of such transformants, mutants were selected for auxotrophy and thermosensitive growth. (**C**) CHEF-type PFGE separated yeast chromosomes from the mutants isolated after AMT and the isogenic wild type.

Accurate DNA replication is a prerequisite for maintenance of genome stability, and any DNA replication stress may be a source of genomic instability and mutation. To find out whether VirD5CT may be mutagenic, we screened for mutation of the *URA3* gene in yeast by selection for 5-fluoroorotic acid (5-FOA) resistance after expression of VirD5CT. In this experiment, we used two yeast strains, each with the *URA3* reporter gene inserted in chromosome III in a different orientation at a short distance of ~1.5 kb from the early replication origin *ARS306* (*37*). This would enable to discriminate the action of VirD5CT from a potential mutagenic effect of the codirectional or head-on collision of the replisome and the transcription machinery. These yeast strains were both transformed with the empty vector pRS425-HYG or the same plasmid carrying a construct that would express VirD5CT from the *GAL1* promoter. Transformants were first grown in YP (yeast extract and peptone)–glucose medium with hygromycin and then shifted to YP-raffinose-galactose medium with hygromycin. After incubation for 6 hours, cells were washed twice with sterile water and then plated onto MY (yeast minimal) medium containing 5-FOA and incubated for 4 days. Thus, it was found that the presence of VirD5CT led to about a 50-fold higher number of 5-FOA–resistant mutants and thus a 50-fold increase in the mutation frequency, irrespective of the orientation of the *URA3* gene (Fig. 5A). We retrieved the *URA3* sequences from a number of these mutants for DNA sequencing and found that they often had small [up to 23 base pairs (bp)] deletions, often bordered by small 2- to 4-bp repeats (fig. S5), which may be indicative of replication slippage (*38*).

### Delivery of VirD5 into yeast during AMT leads to mutations unlinked to the T-DNA

To determine whether the level of VirD5 translocated into host cells during AMT is sufficient to cause mutation in host cells, we compared a pool of yeast transformants that were obtained in four independent cocultivation experiments with an *Agrobacterium* strain wild type for *virD5* (LBA1100) and a similar pool of yeast transformants obtained after four cocultivation experiments with the *virD5* deletion mutant (LBA3551) for the presence of colonies with some easily selectable phenotypes. In this experiment, we arbitrarily chose to select for colonies with one of the four additional requirements for growth (auxotrophic for either lysine, tyrosine, tryptophan, or adenine) and for thermosensitive colonies that do not develop at 37°C. For transformation, binary vector pSDM8001 was used, which contains a G418 resistance gene flanked by *PDA1* sequences to mediate integration by homologous recombination at the *PDA1* locus on chromosome V and thus to avoid insertional mutation other than at this locus by the T-DNA (*39*). We cocultivated haploid yeast BY4741 (*MATa his3*Δ*1 leu2*Δ*0 met15*Δ*0 ura3*Δ*0*) with *Agrobacterium* during 1 week at 21°C and subsequently selected transformants on MY-glucose medium with G418 (200 μg/ml) and cefotaxime (200 μg/ml) to select for the presence of the T-DNA and to kill bacteria, respectively. Besides histidine, leucine, methionine, and uracil, which are all required for growth of BY4741, we additionally added lysine, tyrosine, tryptophan, and adenine to the selection medium to allow growth of putative auxotrophic mutants. The G418-resistant colonies obtained were replica plated twice onto rich medium YPD (yeast extract, peptone, and dextrose) and also twice on MY-glucose minimal medium with only the requirements of BY4741 to be able to find such putative auxotrophic mutants, whereby half of the plates was incubated at 25°C and the other at 37°C to be able to find putative thermosensitive mutants. An outline of the experiment is also depicted in Fig. 5B. An estimated number of about 20,000 G418-resistant transformed colonies were analyzed as described above. All the about 20,000 colonies obtained from the transformation by the *virD5* mutant did grow on all the four different plates. However, among the about 20,000 G418-resistant colonies transformed by wild-type bacteria, 1 was auxotrophic and 9 were thermosensitive, indicating that delivery of VirD5 during AMT was mutagenic in yeast (fig. S6). To exclude that T-DNA had caused any of these mutations by integration at another locus than the *PDA1* locus by nonhomologous end joining, polymerase chain reaction (PCR) was carried out on these mutants to verify whether the T-DNA had inserted at the *PDA1* locus via homologous recombination, which revealed that this was the case in all these lines (fig. S7). However, in lines 28 and 43, only the signature bands for T-DNA integration by homologous recombination were found for the left end of the T-DNA, but not for the right end, suggesting that rearrangements have occurred at the T-DNA integration site in these two lines. To further analyze these strains at the chromosomal level, we separated and visualized the intact chromosomes by PFGE. As can be seen in Fig. 5C, several of the mutants displayed clear size aberrations in single or multiple chromosomes, suggesting that, in the process of AMT, VirD5 had caused gross chromosomal rearrangements (GCRs). When assayed for its growth requirements, the single auxotrophic mutant (number 32 in the figures, also called strain LBYR50) was found to have acquired a lysine auxotrophy. By whole-genome sequencing, it became apparent that this auxotrophy was due to a 1-bp deletion in the *LYS2* gene in chromosome II (fig. S8).

## DISCUSSION

Here, we describe that the expression of VirD5CT in yeast and plant cells strongly inhibits growth. Similar fragmentation of chromosomes was seen after PFGE of chromosomal DNA isolated from yeast cells grown in the presence of VirD5CT as that observed after PFGE of chromosomal DNA isolated from yeast cells treated with a DNA-damaging agent such as bleomycin or hydrogen peroxide (*30*). Growth inhibition by VirD5CT was diminished in cells with multicopy plasmids expressing either of the catalytic subunits of the DNA polymerase α–primase complex. This complex plays an essential role in lagging-strand DNA synthesis during replication, in the initiation of replication at origins of replication, and sometimes in DNA synthesis required for DNA repair. Insufficient expression of the DNA polymerase α–primase *POL1* subunit has previously been shown to lead to replication stress, resulting in the accumulation of ssDNA stretches in the genome, which are vulnerable to mutation and the formation of GCRs (*40*–*42*). Similarly, in yeast expressing VirD5CT, we find much more DNA damage as visualized by foci of Ddc2, which binds to ssDNA stretches bound by RPA for DNA damage signaling, and also foci of Rad52, which represents foci of DNA repair. We also observed that VirD5 increases the mutation frequency in yeast, resulting in localized mutations, especially small deletions, but also in GCRs. Our finding that enhanced expression of the catalytic subunits of the DNA polymerase α–primase complex diminishes the toxicity of VirD5CT indicates that, in the presence of VirD5CT, the yeast cell has a higher demand for DNA polymerase α–primase. This may be because of replication stress, leading to ssDNA gaps and/or DNA breaks that must be repaired by homologous recombination, requiring new DNA synthesis, or requiring new origins of replication to be activated to rescue stalled forks.

How VirD5 causes replication stress and DNA damage is unknown, but the observation that VirD5 is toxic not only to yeast but also to mammalian and plant cells (*22*, *23*) suggests that VirD5 interacts with important conserved host factors. One of the known VirD5 interactors is the transcription elongation factor Spt4 (*23*). We found that the growth of an *spt4* mutant is much less inhibited by VirD5 (*23*) or VirD5CT (this study) than the wild type, which indicates that interaction with Spt4 is critical for the toxicity of VirD5. The Spt4 protein is a transcription elongation factor, which also has a hitherto poorly described function at the centromeres/kinetochores of the chromosomes (*27*). Previously, we showed that retrieval of VirD5 via Spt4 to the centromeres/kinetochores leads to further interactions of the N-terminal domain with kinetochore components (*23*, *28*). Through its N-terminal domain, which includes a repetitive structure (*28*), VirD5 hyperactivates Aurora kinase at the centromeres, causing chromosome missegregation during mitosis, leading to chromosome loss and aneuploidy (*23*, *28*). The toxicity of VirD5CT, which does not form foci at the centromeres/kinetochores, is also dependent on the presence of Spt4. This suggests that VirD5CT (and, by inference, also full VirD5) may also interact with Spt4, when Spt4 is part of the transcriptional machinery. By affecting transcription, it may either increase the likelihood of collisions between the transcription and the replication machinery or lead to other replication blocks such as the formation of R loops (*43*). This, in turn, would lead to replication stress, replication fork stalling, formation of ssDNA gaps, and, if repair is not occurring in a timely manner, fork collapse and double-strand break formation (*35*). Recently, *Agrobacterium* infection was shown to induce the expression of DNA damage response and DNA repair genes in *A. thaliana*, and this was not dependent on the T-DNA but on the presence of the *Agrobacterium* virulence system (*44*). In view of our results, VirD5-induced DNA damage may have been responsible for the up-regulation of these genes in plants.

Increasing replication stress due to growth in the presence of HU and also mutations in specific DNA repair genes make yeast cells more sensitive to VirD5CT. Inactivation of genes, which play a crucial role in fork protection and repair (*36*), such as *RAD51* and *RAD52*, leads to yeast cells that are hypersensitive to VirD5. This is also the case for *srs2* and *rad9* mutants. The *SRS2* gene encodes a helicase that dismantles Rad51 nucleoprotein filaments, which is important during gene conversion repair (*45*). The *RAD9* gene encodes a protein with similarity to the mammalian 53BP1 protein (*46*) and, along with the 9-1-1 complex, is involved in coordination of the DNA damage checkpoint. However, the absence of the genes coding for the 9-1-1 complex does not aggravate the susceptibility of yeast cells to VirD5. This suggests that the susceptibility of *rad9* cells to VirD5 is a due to the loss of a second function of Rad9, namely, the protection of stalled replication forks from exonuclease degradation (*47*). Last, the absence of the genes coding for the MRX complex (*MRE11*, *RAD50*, and *XRS2*), which is required for fork protection and postreplication repair (*48*), increased the susceptibility of yeast to VirD5.

In the absence of VirD5, the transformation frequency is decreased (*26*). Why this is the case is unknown, but our finding that VirD5 causes DNA damage and DNA breaks suggests that VirD5 may form entry points for T-DNA integration. However, VirD5 also has several other properties with which it could support transformation. VirD5 can act as a transcription factor that binds to a specific sequence in the promoter regions of some genes (*25*, *49*). However, these genes differ from those found to be induced by *Agrobacterium* infection (*44*). It has also been published that VirD5 not only may promote decoating of the T-complex by stabilizing the VirF protein (*24*) but also, reversely, may compete with VBF for interacting with VIP1, thus preventing the decoating of the T-complex (*25*). Last, VirD5 was found to compete with the host cap-binding protein CBP20 for binding to the host protein VIP2 (*26*). VirD5 is a large protein with 833 amino acids (*17*), which is conserved in all *Agrobacterium* strains isolated so far and may therefore contribute to the transformation process in several ways.

A feature of T-DNA–transformed plant cells is that they can contain mutations not linked to T-DNA integration (*12*–*15*). Such mutations have mostly been attributed to somaclonal variation resulting from plant cell and tissue culture, which may lead to an about 250-fold higher base substitution rate (*14*, *15*). However, also after floral dip transformation, which does not involve tissue culture steps, mutations not linked to the T-DNA have been observed (*14*, *15*). Analysis of five transgenic lines obtained after floral dip by NGS found five base substitutions and five 2- to 5-bp deletions, indicating a mutation frequency not different from the mutation frequency in seed-propagated plants (*14*). However, after a direct comparison by NGS of a larger pool of rice plants regenerated from tissue culture with or without *Agrobacterium*, it became apparent that the numbers of single-nucleotide variants were similar but that there were two- to threefold more indels after *Agrobacterium* infection (*50*). In analyzing the mutation of the *URA3* gene, we found that the expression of VirD5 in yeast induced small deletions with a signature of replication slippage in particular. This mutational pattern is different from what is seen with spontaneous mutations in this gene (*51*). The mutational spectrum of 207 spontaneous mutations consisted mostly of base substitutions (167) and single-base insertions or deletions (*28*) but showed no larger deletions as we found after expression of VirD5 (*51*). Together, this suggests that VirD5 delivery into host cells during transformation is unlikely to strongly increase the mutation frequency but is still better avoided, due to the signature of the elicited mutations, i.e., small deletions that can more easily inactivate a gene than base substitutions that are the most common spontaneous mutations. Deletion mutation by VirD5 may also explain that of the 16 mutants lacking seed coat pigmentation isolated from a pool of T-DNA–transformed lines, only 7 had a T-DNA–tagged mutation, while 6 had a small (3 to 56 bp) deletion (*15*). In addition, there was a large deletion in one line and a base substitution leading to a missense mutation in two lines (*15*).

To find out whether VirD5 is mutagenic during AMT, we compared yeast colonies transformed by either the wild type or the *virD5* mutant. In this way, we found that, in the pool of about 20,000 yeast colonies transformed by the wild type, 9 thermosensitive colonies and 1 colony auxotrophic for lysine were present, but such mutants were not found in a similar number of yeast colonies transformed by the virD5 mutant. Although this frequency seems extremely small, we have to consider that most of the putative mutations induced by VirD5 will not generate the arbitrarily selected phenotypes we selected for, and further work needs to be done to establish the mutation frequency. Such mutations are disadvantageous when applying AMT for biotechnological purposes, as well as in basic research when studies of mutants from collections of T-DNA insertion mutants, “unexpectedly” reveal that a mutational phenotype is not linked to the T-DNA. Our results suggest that the number of unlinked mutations associated with T-DNA transformation and especially the formation of small deletions can be reduced by using an *Agrobacterium virD5* mutant instead of the wild-type helper strain for genome engineering, although this may also lead to a lower transformation frequency.

In this work, we have used yeast as a model to study why the *Agrobacterium* virulence protein VirD5 is toxic to eukaryotic cells. It remains to be seen whether the toxicity of VirD5 in plant cells is due to the same molecular mechanism as demonstrated here for yeast. This is plausible, however, as VirD5 interacts with the two AtSpt4 orthologs that are present in *A. thaliana* (*23*).

## MATERIALS AND METHODS

### Primers, strains, and plasmids

Primers, yeast strains, and plasmids used in this study are listed in tables S1 to S3, respectively. The yeast deletion mutants used were in the diploid BY4743 background and obtained from EUROSCARF. For labeling of *RAD52* and *DDC2* with GFP, strain W303-1A was transformed with a DNA fragment obtained by PCR using plasmids pYM27 with eGFP-KanMX (EUROSCARF) and pJET1.2 with 3xGFP-KanMX (Thermo Fisher Scientific) as a template, respectively, as previously described (*52*). *Agrobacterium* strains LBA1100 (*7*) and LBA3551, which is LBA1100Δ*virD5*, were used for transformation.

### Plant experiments

Binary vector pGPINTAM-VirD5CT (521 to 833 amino acids) containing *virD5CT* under the control of a tamoxifen-inducible promoter was transferred into *A. tumefaciens* strain AGL1 (*53*). *A. thaliana* ecotype Columbia-0 was used for floral dip. A few weeks after dipping, mature seeds were harvested and sown on MS (Murashige and Skoog) medium containing kanamycin (50 mg/liter). Kanamycin-resistant T1 transgenic seedlings were checked for the insert by PCR and transferred to soil. T2 seeds from eight independent T1 transgenic plants were germinated on MS medium containing kanamycin and either dimethyl sulfoxide or 1 μM tamoxifen to induce the expression of VirD5CT. All these lines were sensitive to the expression of VirD5CT. Two of these lines are shown in Fig. 1.

### Visualization of GFP-labeled proteins

Plasmids expressing GFP-labeled proteins were transformed into yeast BY4743 cells. Transformants were grown at 30°C on solid medium under conditions that suppress the expression of VirD5 and its derivatives. Three days after transformation, colonies were transferred to similar liquid medium. Overnight cultures were diluted and grown at 30°C in fresh liquid medium under conditions that induce the expression of VirD5 (derivatives). GFP signal (excitation, 488 nm; emission, 520 nm) was visualized using a 63× oil objective on the Zeiss Imager confocal microscope. Images were processed with ImageJ (ImageJ National Institutes of Health) and Photoshop (Adobe). To promote nuclear import of VirD5CT (labeled with GFP), the NLS (PKKKRKV; encoded by primers SV40F and SV40R, table S3) from the SV40 large tumor antigen (SV40 large T antigen) was fused at the C terminus of VirD5CT. To promote nuclear export, the NES (LQLPPLERLTLGA; encoded by primer VirD5#86, table S3) from the HIV Rev protein was fused at the N terminus of VirD5CT.

### Separation of chromosomes by CHEF technology–based PFGE

Wild-type BY4743 and cells with a chromosomally integrated copy of *virD5CT* driven by the *GAL1* promoter were cultured in a YP [yeast extract (10 g/liter) and peptone (20 g/liter)]–rich medium containing 2% glucose. Overnight cultured cells were diluted to an OD_620_ (optical density at 620 nm) of 0.1 and then cultured in YP-rich medium containing 2% raffinose for additional 6 hours, followed by addition of 2% galactose and cultivation overnight.

Intact chromosomes from overnight cultured yeast cells were isolated in agarose plugs as described in the CHEF kit (Bio-Rad, 170-3591). A number of 6 × 10^8^ yeast cells were washed twice with 0.1 M EDTA (pH 7.5) and resuspended in 630 μl of suspension buffer. The suspension mixed with 370 μl of 2% low-melt agarose was used to make plugs for CHEF technology–based PFGE. Plugs were placed in a 1% agarose gel and sealed with liquid agarose. Electrophoresis was carried out in 0.5× Tris-borate-EDTA at 14°C for 24 hours with an initial switch time of 60 s and a final switch time of 90 s at 200 V. The separated chromosomes were stained with ethidium bromide.

### Genome-wide overexpression assay

The plasmid (pMVHis-NES-VirD5CT) encoding NES-VirD5CT under the control of the *GAL1* promoter was transformed either with a high-copy number pRS425 or with the plasmids of a yeast genomic tiling collection (*31*) consisting of around 1500 individual plasmids with about 10–kilo-bp (kbp) inserts of yeast genomic DNA (YSC4613, Dharmacon, USA) into wild-type BY4743 cells. On average, each genomic DNA insertion contains four to five genes with their own promoters and terminators. Transformants were selected on MY medium (*52*) containing 2% glucose and histidine. Three days after growing on MY-glucose plates, colonies were suspended in H_2_O and spotted onto MY medium containing 2% galactose. Plasmids from the survived colonies were mapped according to the Yeast Genomic Tiling Collection and were retransformed with pMVHis-NES-VirD5CT into BY4743 cells. After 3 days of growing on MY-glucose plates, colonies were suspended, fivefold diluted, and spotted onto MY medium containing either 2% glucose or 2% galactose.

### Measurement of *ura3* mutation rates

To measure the mutation rates at the *URA3* locus, we used strains, which have the *URA3* reporter gene integrated either in orientation 1 (yTAK001) or orientation 2 (yTAK002) at the *AGP1* locus, which is 1.5 kbp from the early replication origin ARS306 in chromosome III (*37*). These yeast strains were transformed either with the single-copy vector containing a hygromycin resistance gene (pRS315-HYG) or the same vector encoding VirD5CT under the control of the *GAL1* promoter (pRS315-HYG-GAL1-VirD5CT). Transformants were selected on YP-rich agar medium containing 2% glucose and hygromycin (200 μg/ml) and incubated for 3 days at 30°C. Positive colonies were inoculated in YP liquid medium containing 2% glucose and hygromycin (200 μg/ml) overnight. Overnight cultured cells were diluted to an OD_620_ of 0.1 and recultured in YP-rich medium containing 2% raffinose and hygromycin (200 μg/ml) for an additional 6 hours, followed by addition of 2% galactose and cultivation for another 6 hours. Cells were diluted to appropriate concentrations and plated onto either YP medium containing 2% glucose and hygromycin (200 μg/ml) for output or onto MY medium containing 2% glucose, hygromycin (200 μg/ml), and 5-FOA for selection of mutants. Plates were incubated at 30°C for 2 days (YP) or 4 days (MY). The mutation frequencies were calculated by dividing the number of 5-FOA–resistant colonies by the total number of yeast cells.

### AMT of yeast

AMT was performed according to our previously published protocol (*7*) with some modifications. Briefly, wild-type *Agrobacterium* LBA1100 (pSDM8001) and the *virD5* mutant LBA3551 (pSDM8001) were grown overnight in LB medium with rifamycin and kanamycin. Overnight cultured bacteria were diluted to OD_600_ = 0.25 and recultured in induction medium (IM) containing 200 μM acetosyringone for an additional 6 hours at 28°C. The yeast strain BY4741 (*MATa his3*Δ1 *leu2*Δ0 *met15*Δ0 *ura*3Δ0) was cultured overnight in YPD [yeast extract (10 g/liter), peptone (20 g/liter), and d-glucose (20 g/liter)]. Overnight cultured yeast cells were diluted to OD_620_ = 0.1 and recultured in YPD medium for additional 5 to 6 hours. The *Agrobacterium* cells were washed with IM without glucose before mixing with the yeast cells. *Agrobacterium*-yeast cocultivations were incubated for 7 days at 21°C on filter on IM containing four supplements (histidine, leucine, methionine, and uracil), which are needed by BY4741 for growth, and four extra supplements (lysine, tyrosine, tryptophan, and adenine) to allow growth of putative mutants with mutations giving a requirement for any of these four supplements. The mixtures were suspended in 0.9% NaCl; plated on MY medium containing cefotaxime (200 μg/ml), G418 (200 μg/ml), and all the eight supplements; and then incubated at 25°C for 4 days. Plates with transformant colonies were replicated onto two YPD plates with G418, which were incubated at either 25° or 37°C. Temperature-sensitive mutants were found as colonies that were growing on plates incubated at 25°C, but not on those incubated at 37°C (green spot in Fig. 5B). At the same time, colonies were also replica plated on MY with G418 and contained only the four supplements (histidine, leucine, methionine, and uracil) required by BY4741 for growth, but not any of the four additional supplements that were present during the cocultivation with *Agrobacterium*. These plates were also incubated at 25° or 37°C. Colonies growing on YPD-rich medium, but not on these MY plates, would represent auxotrophic mutants (white spot on the MY plates in Fig. 5B). Putative auxotrophic or temperature-sensitive mutants were isolated and further analyzed.

### Whole-genome sequencing of the auxotrophic yeast strain

Genomic DNA was isolated from the auxotrophic strain and from wild-type BY4741 using QIAGEN Genomic-Tip 100/G gravity-flow columns. Illumina NovaSeq paired-end sequencing was performed at BaseClear (Leiden, The Netherlands). Following quality filtering and adapter removal, reads were mapped to the BY4741_Toronto_2012 genome sequence from the Saccharomyces Genome Database using BWA-MEM (*54*). BAM files were sorted and duplicate reads were marked with SAMtools and Picard v2.20.0 MarkDuplicates, respectively. Variants were called using FreeBayes v1.2.0 (*55*). Variants were filtered using the vcffilter utility from vcflib based on the following criteria: QUAL > 1, QUAL/AO > 10, SAF > 0, SAR > 0, RPR > 1, and RPL > 1. A single variant was specific for the auxotrophic strain. It was a 1-bp deletion in the *LYS2* gene. Sequencing reads were uploaded to the Sequence Read Archive (SRA) under BioProject PRJNA834975.

## Supplementary Materials

### This PDF file includes:

Figs. S1 to S8 Tables S1 to S3 References

## Notes

View/request a protocol for this paper from *Bio-protocol*.
