# Supplementary material for: The translocated virulence protein VirD5 causes DNA damage and mutation during Agrobacterium-mediated transformation of yeast
Source: Sci Adv. 2022 Nov 16;8(46):eadd3912. doi: 10.1126/sciadv.add3912 (PMC9668295; doi:10.1126/sciadv.add3912)
Supplement: Supplementary file 1 — Figs. S1 to S8 Tables S1 to S3 References [file sciadv.add3912_sm.pdf]

Science Advances
AAAS

Supplementary Materials for

The translocated virulence protein VirD5 causes DNA damage and mutation during *Agrobacterium*-mediated transformation of yeast

Xiaorong Zhang *et al.*

Corresponding author: Paul J. J. Hooykaas, P.J.J.Hooykaas@biology.leidenuniv.nl

Sci. Adv. **8**, eadd3912 (2022)
DOI: 10.1126/sciadv.add3912

This PDF file includes:

Figs. S1 to S8
Tables S1 to S3
References

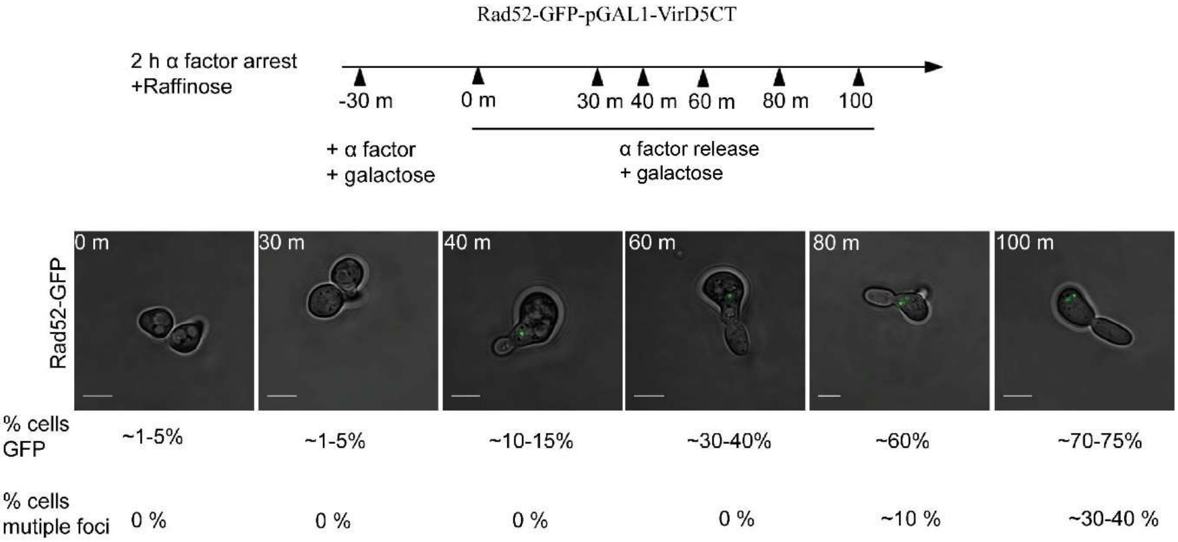

Figure S1. VirD5CT causes DNA damage in yeast.
DNA damage was visualized by the formation of Rad52-GFP DNA repair foci in yeast cells expressing VirD5CT from the *GAL1* promoter. Cells were arrested by α factor for 2 hours. Expression of VirD5CT was induced by the addition of galactose 30 minutes before release from arrest. Repair foci start to accumulate from about 40 minutes after release from α factor arrest.

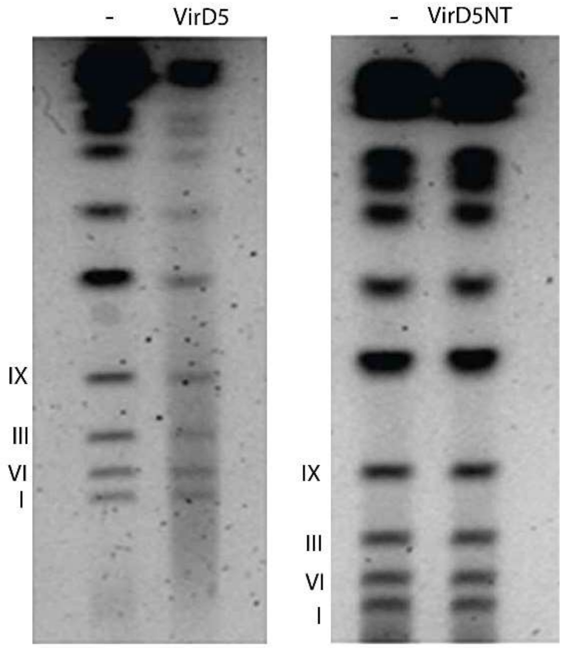

Figure S2. Visualization of the yeast chromosomes after PFGE.
(Left PFGE gel) Comparison of chromosomes from wild-type cells with cells expressing complete VirD5. (Right PFGE gel) Comparison of those from yeast expressing GFP with yeast expressing GFP-VirD5NT. GFP-VirD5NT is equally toxic as VirD5NT without GFP tag.

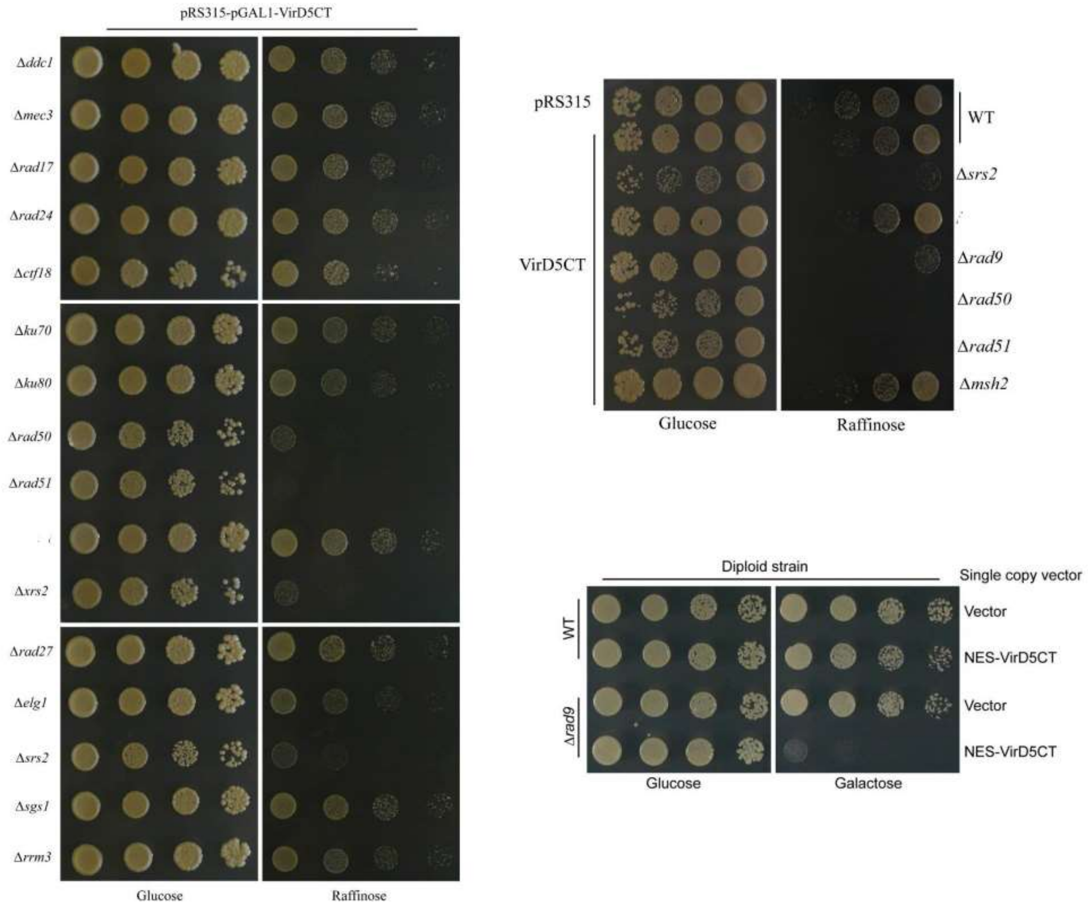

Figure S3. Susceptibility of various DNA repair mutants to the expression of VirD5CT. The growth inhibitory effect of a low amount of VirD5CT present in raffinose cultured cells is exacerbated in some, but not all mutants. For the *rad9* mutant, growth inhibition in the presence of NES-VirD5CT is also shown in the lower panel on the right.

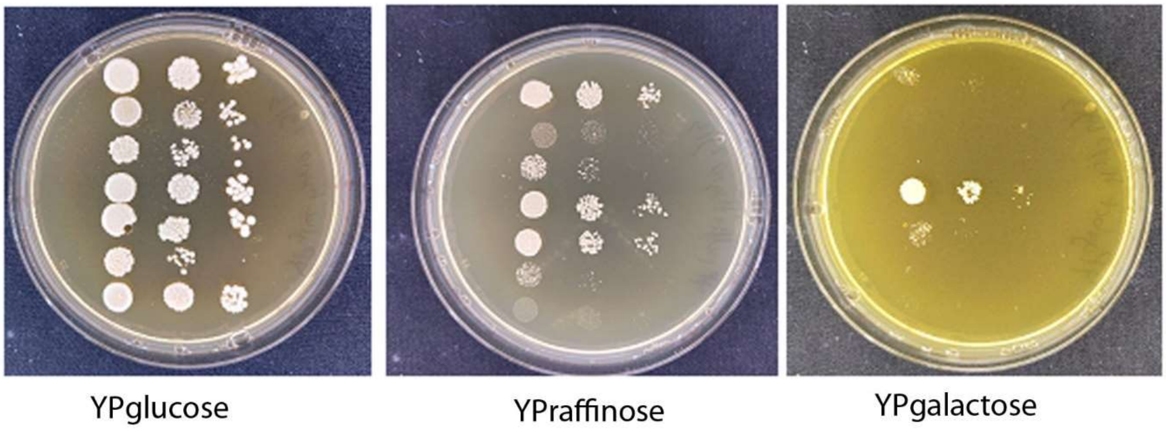

Figure S4. Yeast cells with an *spt4* deletion are less susceptible to the inhibitory effects of VirD5CT than wild-type cells.

Growth on glucose (left; no VirD5 expression), raffinose (middle; low VirD5CT expression), and galactose (right; high VirD5CT expression). From top to bottom: dilutions of wild-type BY4343 yeast cells, followed by mutants for *mre11*, *xrs2*, *spt4*, *rad50*, *rad51*, and *rad52*. On galactose only the *spt4* mutant shows growth.

```
36_20F9ZAF028.ab1                                   CAAACAAACTTGT-GTGCTTCATTGGATGTTCGT
36_20F9ZAF024.ab1                                   CAAA-AGCTTTGTCGTGCTTCATTGGATGTTCGT
36_20F9ZAF023.ab1                                   CAAAAAACTTAGTAGTGCTTCATTGGATGTTCGT
36_20F9ZAF069.ab1                                   CAAACAAACTTGT-GTGCTTCATTGGATGTTCGT
36_20F9ZAF071.ab1                                   CAAACAAACTTGT-GTGCTTCATTGGATGTTCGT
URA3                                                CAAACAAACTTGT-GTGCTTCATTGGATGTTCGT
39_20F9ZAF076.ab1                                   CAAACAAACTTGT-GTGCTTCATTGGATGTTCGT
39_20F9ZAF033.ab1                                   CAAACAAACTTGT-GTGCTTCATTGGATGTTCGT
39_20F9ZAF032.ab1                                   CAAACAAACTTGT-GTGCTTCATTGGATGTTCGT
39_20F9ZAF077.ab1                                   CAAACAAACTTGT---------------------
39_20F9ZAF075.ab1                                   CAAACAAACTTGT-GTGCTTCATTGGATGTTCGT
39_20F9ZAF038.ab1                                   CAAACAAACTTGT-GTGCTTCATTGGATGTTCGT
LBYR36_20F9ZAF068.ab1                               CAAACAAACTTGT-GTGCTTCATTGGATGTTCGT

36_20F9ZAF028.ab1          ACCACCAAGGAATTACTGGAGTTAGTTGAAGCATTAGGTCCCAAAATTTGTTTACTAAAA
36_20F9ZAF024.ab1          ACCACCAAGGAATTACTGGAGTTAGTTGAAGCATTAGGTCCCAAAATTTGTTTACTAAAA
36_20F9ZAF023.ab1          ACCACCAAGGAATTACTGGAGTTAGTTGAAGCATTAGGTCCCAAAATTTGTTTACTAAAA
36_20F9ZAF069.ab1          ACCACCAAGGAATTACTGGAGTTAGTTGAAGCATTAGGTCCCAAAATTTGTTTACTAAAA
36_20F9ZAF071.ab1          ACCACCAAGGAATTACTGGAGTTAGTTGAAGCATTAGGTCCCAAAATTTGTTTACTAAAA
URA3                       ACCACCAAGGAATTACTGGAGTTAGTTGAAGCATTAGGTCCCAAAATTTGTTTACTAAAA
39_20F9ZAF076.ab1          ACCACCAAGGAATTACTGGAGTTAGTTGAAGCATTAGGTCCCAAAATTTGTTTACTAAAA
39_20F9ZAF033.ab1          ACCACCAAGGAATTACTGGAGTTAGTTGAAGCATTAGGTCCCAAAATTTGTTTACTAAAA
39_20F9ZAF032.ab1          ACCACCAAGGAATTACTGGAGTTAGTTGAAGCATTAGGTCCCAAAATTTGTTTACTAAAA
39_20F9ZAF077.ab1          ACCACCAAGGAATTACTGGAGTTAGTTGAAGCATTAGGTCCCAAAATTTGTTTACTAAAA
39_20F9ZAF075.ab1          ACCACCAAGGAATTACTGGAGTTAGTTGAAGCATTAGGTCCCAAAATTTGTTTACTAAAA
39_20F9ZAF038.ab1          ACCACCAAGGAATTACTGGAGTTAGTTGAAGCATTAGGTCCCAAAATTTGTTTACTAAAA
LBYR36_20F9ZAF068.ab1      ACCACCAAGGAATTACTGGAGTTAGTTGAAGCATTAGGTCCCAAAATTTGTTTACTAAAA

36_20F9ZAF028.ab1          ACACATGTGGATATCTTGACTGATTTTTCCATGGAGGGCACAGTTAAGCCGCTAAAGGCA
36_20F9ZAF024.ab1          ACACATGTGGATATCTTGACTGATTTTTCCATGGAGGGCACAGTTAAGCCGCTAAAGGCA
36_20F9ZAF023.ab1          ACACATGTGGATATCTTGACTGATTTTTCCATGGAGGGCACAGTTAAG---------GCA
36_20F9ZAF069.ab1          ACACATGTGGATATCTTGACTGATTTTTCCATGGAGGGCACAGTTAAGCCGCTAAAGGCA
36_20F9ZAF071.ab1          ACACATGTGGATATCTTGACTGATTTTTCCATGGAGGGCACAGTTAAGCCGCTAAAGGCA
URA3                       ACACATGTGGATATCTTGACTGATTTTTCCATGGAGGGCACAGTTAAGCCGCTAAAGGCA
39_20F9ZAF076.ab1          ACACATGTGGATATCTTGACTGATTTTTCCATGGAGGGCACAGTTAAGCCGCTAAAGGCA
39_20F9ZAF033.ab1          ACACATGTGGATATCTTGACTGATTTTTCCATGGAGGGCACAGTTAAGCCGCT-------
39_20F9ZAF032.ab1          ACACATGTGGATATCTTGACTGATTTTTCCATGGAGGGCACAGTTAAGCCGCTAAAGGCA
39_20F9ZAF077.ab1          ACACATGTGGATATCTTGACTGATTTTTCCATGGAGGGCACAGTTAAGCCGCTAAAGGCA
39_20F9ZAF075.ab1          ACACATGTGGATATCTTGACTGATTTTTCCATGGAGGGCACAGTTAAGCCGCTAAAGGCA
39_20F9ZAF038.ab1          ACACATGTGGATATCTTGACTGATTTTTCCATGGA-GGCACAGTTAAGCCGCTAAAGGCA
LBYR36_20F9ZAF068.ab1      ACACATGTGGATATCTTGACTGATTTTTCCATGGAGGGCACAGTTAAGCCGCTAAAGGCA

36_20F9ZAF028.ab1          TTATCCGCCAAGTACAATTTTTTACTCTTCTAAGACAGAAAATTTGCTGACATTGGTAAT
36_20F9ZAF024.ab1          TTATCCGCCAAGTACAATTTTTTACTCTTCGAAGACAGAAAATTTGCTGACATTGGTAAT
36_20F9ZAF023.ab1          TTATCCGCCAAGTACAATTTTTTACTCTTCGAAGACAGAAAATTTGCTGACATTGGTAAT
36_20F9ZAF069.ab1          TTATCCGCCAAGTACAATTTTTTACTCTTCGAAGACAGAAAATTTGCTGACATTGGTAAT
36_20F9ZAF071.ab1          TTATCCGCCAAGTACAATTTTTTACTCTTCGAAGACAGAAAATTTGCTG-----------
URA3                       TTATCCGCCAAGTACAATTTTTTACTCTTCGAAGACAGAAAATTTGCTGACATTGGTAAT
39_20F9ZAF076.ab1          TTATCCGCCAAGTACAATTTTTTACTCTTCGAAGACAGAAAATTTGCTGACATTGGTAAT
39_20F9ZAF033.ab1          ---------AAGTACAATTTTTTACTCTTCGAAGACAGAAAATTTGCTGACATTGGTAAT
39_20F9ZAF032.ab1          TTATCCGCCAAGTACAATTTTTTACTCTTCGAAGACAGAAAATTTGCTGACATTGGTAAT
39_20F9ZAF077.ab1          TTATCCGCCAAGTACAATTTTTTACTCTTCGAAGACAGAAAATTTGCTGACATTGGTAAT
39_20F9ZAF075.ab1          TTATCCGCCAAGTACAATTTTTTACTCTTCGAAGACAGAAAATTTGCTGACATTGGTAAT
39_20F9ZAF038.ab1          TTATCCGCCAAGTACAATTTTTTACTCTTCGAAGACAGAAAATTTGCTGACATTGGTAAT
LBYR36_20F9ZAF068.ab1      TTATCCGCCAAGTACAATTTTTTACTCTTCGAAGACAGAAAATTTGCTGACATTGGTAAT

36_20F9ZAF028.ab1          ACAGTCAAATTGCAGTACTCTGCGGGTGTATACAGAATAGCAGAATGGGCAGACATTACG
36_20F9ZAF024.ab1          ACAGTCAAATTGCAGTACTCTGCGGGTGTATACAGAATAGCAGAATGGGCAGACATTACG
36_20F9ZAF023.ab1          ACAGTCAAATTGCAGTACTCTGCGGGTGTATACAGAATAGCAGAATGGGCAGACATTACG
36_20F9ZAF069.ab1          ACAGTCAAATTGCAGTACTCTGCGGGTGTATACAGAATAGCAGAATGGGCAGACATTACG
36_20F9ZAF071.ab1          ACAGTCAAATTGCAGTACTCTGCGGGTGTATACAGAATAGCAGAATGGGCAGACATTACG
URA3                       ACAGTCAAATTGCAGTACTCTGCGGGTGTATACAGAATAGCAGAATGGGCAGACATTACG
39_20F9ZAF076.ab1          ACAGTCAAATTGCAGTACTCTGC-----------------------GGGCAGACATTACG
39_20F9ZAF033.ab1          ACAGTCAAATTGCAGTACTCTGCGGGTGTATACAGAATAGCAGAATGGGCAGACATTACG
39_20F9ZAF032.ab1          ACAGTCAAATTGCAGTACTCTGCGGGTGTATACAGAATAGCAGAATGGGCAGACATTACG
39_20F9ZAF077.ab1          ACAGTCAAATTGCAGTACTCTGCGGGTGTATACAGAATAGCAGAATGGGCAGACATTACG
39_20F9ZAF075.ab1          ACAGTCAAATTGCAGTACTCTGCGGGTGTATACAGAATAGCAGAATGGGCAGACATTACG
39_20F9ZAF038.ab1          ACAGTCAAATTGCAGTACTCTGCGGGTGTATACAGAATAGCAGAATGGGCAGACATTACG
LBYR36_20F9ZAF068.ab1      ACAGTCAAATTGCAGTACTCTGCGGGTGTATACAGAATAGCAGAATGGGCAGACATTACG
```

```
36_20F9ZAF028.ab1          AATGCACACGGTGTGGTGGGCCCAGGTATTGTTAGCGGTTTGAAGCAGGCGGCAGAAGAA
36_20F9ZAF024.ab1          AATGCACACGGTGTGGTGGGCCCAGGTATTGTTAGCGGTTTGAAGCAGGCGGCAGAAGAA
36_20F9ZAF023.ab1          AATGCACACGGTGTGGTGGGCCCAGGTATTGTTAGCGGTTTGAAGCAGGCGGCAGAAGAA
36_20F9ZAF069.ab1          AATGCACACGGTGTGGTGGGCCCAGGTATTGTTAGCGGTTTGAAGCAGGCGGCAGAAGAA
36_20F9ZAF071.ab1          AATGCACACGGTGTGGTGGGCCCAGGTATTGTTAGCGGTTTGAAGCAGGCGGCAGAAGAA
URA3                       AATGCACACGGTGTGGTGGGCCCAGGTATTGTTAGCGGTTTGAAGCAGGCGGCGGAAGAA
39_20F9ZAF076.ab1          AATGCACACGGTGTGGTGGGCCCAGGTATTGTTAGCGGTTTGAAGCAGGCGGCAGAAGAA
39_20F9ZAF033.ab1          AATGCACACGGTGTGGTGGGCCCAGGTATTGTTAGCGGTTTGAAGCAGGCGGCAGAAGAA
39_20F9ZAF032.ab1          AATGCACACGGTGTGGTGGGCC-----------------------CAGGCGGCAGAAGAA
39_20F9ZAF077.ab1          AATGCACACGGTGTGGTGGGCCCAGGTATTGTTAGCGGTTTGAAGCAGGCGGCAGAAGAA
39_20F9ZAF075.ab1          AATGCACACGGTGTGGTGGGCCCAGGTATTGTTAGCGGTTTGAAGCAGGCGGCAGAAGAA
39_20F9ZAF038.ab1          AATGCACACGGTGTGGTGGGCCCAGGTATTGTTAGCGGTTTGAAGCAGGCGGCAGAAGAA
LBYR36_20F9ZAF068.ab1      AATGCACACGGTGTGGTGGGCCCAGGTATTGTTAGCGGTTTGAAGCAGGCGGCAGAAGAA

36_20F9ZAF028.ab1          GTAACAAAGGAACCTAGAGGCCTTTTGATGTTAGCAGAATTGTCATGCAAGGGCTCCCTA
36_20F9ZAF024.ab1          GTAACAAAGGAACCTAGAGGCCTTTTGATGTTAGCAGAATTGTCATGCAAGGGCTCCCTA
36_20F9ZAF023.ab1          GTAACAAAGGAACCTAGAGGCCTTTTGATGTTAGCAGAATTGTCATGCAAGGGCTCCCTA
36_20F9ZAF069.ab1          GTAACAAAGGAACCTAGAGGCCTTTTGATGTTAGCAGAATTGTCATGCAAGGGCTCCCTA
36_20F9ZAF071.ab1          GTAACAAAGGAACCTAGAGGCCTTTTGATGTTAGCAGAATTGTCATGCAAGGGCTCCCTA
URA3                       GTAACAAAGGAACCTAGAGGCCTTTTGATGTTAGCAGAATTGTCATGCAAGGGCTCCCTA
39_20F9ZAF076.ab1          GTAACAAAGGAACCTAGAGGCCTTTTGATGTTAGCAGAATTGTCATGCAAGGGCTCCCTA
39_20F9ZAF033.ab1          GTAACAAAGGAACCTAGAGGCCTTTTGATGTTAGCAGAATTGTCATGCAAGGGCTCCCTA
39_20F9ZAF032.ab1          GTAACAAAGGAACCTAGAGGCCTTTTGATGTTAGCAGAATTGTCATGCAAGGGCTCCCTA
39_20F9ZAF077.ab1          GTAACAAAGGAACCTAGAGGCCTTTTGATGTTAGCAGAATTGTCATGCAAGGGCTCCCTA
39_20F9ZAF075.ab1          GTAACAAAGGAACCTAGAGGCCTTTTGATGTTAGCAGAATTGTCATGCAAGGGCTCCCTA
39_20F9ZAF038.ab1          GTAACAAAGGAACCTAGAGGCCTTTTGATGTTAGCAGAATTGTCATGCAAGGGCTCCCTA
LBYR36_20F9ZAF068.ab1      GTAACAAAGGAACCTAGAGGCCTTTTGATGTTAGCAGAATTGTCATGCAAGGGCTCCCTA

36_20F9ZAF028.ab1          TCTACTGGAGAATATACTAAGGGTACTGTTGACATTGCGAAGAGCGACAAAGATTTTGTT
36_20F9ZAF024.ab1          TCTACTGGAGAATATACTAAGGGTACTGTTGACATTGCGAAGAGCGACAAAGATTTTGTT
36_20F9ZAF023.ab1          TCTACTGGAGAATATACTAAGGGTACTGTTGACATTGCGAAGAGCGACAAAGATTTTGTT
36_20F9ZAF069.ab1          TCTACTGGAGAATATACTAAGGGTACTGTTGACATTGCGAAGAGCGACAAAGATTTTGTT
36_20F9ZAF071.ab1          TCTACTGGAGAATATACTAAGGGTACTGTTGACATTGCGAAGAGCGACAAAGATTTTGTT
URA3                       GCTACTGGAGAATATACTAAGGGTACTGTTGACATTGCGAAGAGCGACAAAGATTTTGTT
39_20F9ZAF076.ab1          TCTACTGGAGAATATACTAAGGGTACTGTTGACATTGCGAAGAGCGACAAAGATTTTGTT
39_20F9ZAF033.ab1          TCTACTGGAGAATATACTAAGGGTACTGTTGACATTGCGAAGAGCGACAAAGATTTTGTT
39_20F9ZAF032.ab1          TCTACTGGAGAATATACTAAGGGTACTGTTGACATTGCGAAGAGCGACAAAGATTTTGTT
39_20F9ZAF077.ab1          TCTACTGGAGAATATACTAAGGGTACTGTTGACATTGCGAAGAGCGACAAAGATTTTGTT
39_20F9ZAF075.ab1          TCTACTGGAGAATATACTAAGGGTACTGTTGACATTGCGAAGAGCGACAAAGATTTTGTT
39_20F9ZAF038.ab1          TCTACTGGAGAATATACTAAGGGTACTGTTGACATTGCGAAGAGCGACAAAGATTTTGTT
LBYR36_20F9ZAF068.ab1      TCTACTGGAGAATATACTAAGGGTACTGTTGACATTGCGAAGAGCGACAAAGATTTTGTT

36_20F9ZAF028.ab1          ATCGGCTTTATTGCTCAAAGAGACATGGGT-GGAAGAGATGAAGGTTACGATTGGTTGAT
36_20F9ZAF024.ab1          ATCGGCTTTATTGCTCAAAGAGACATGGGT-GGAAGAGATGAAGGTTACGATTGGTTGAT
36_20F9ZAF023.ab1          ATCGGCTTTATTGCTCAAAGAGACATGGGT-GGAAGAGATGAAGGTTACGATTGGTTGAT
36_20F9ZAF069.ab1          ATCGGCTTTATTGCTCAAAGAGACATGGGTGGGAAGAGATGAAGGTTACGATTGGTTGAT
36_20F9ZAF071.ab1          ATCGGCTTTATTGCTCAAAGAGACATGGGT-GGAAGAGATGAAGGTTACGATTGGTTGAT
URA3                       ATCGGCTTTATTGCTCAAAGAGACATGGGT-GGAAGAGATGAAGGTTACGATTGGTTGAT
39_20F9ZAF076.ab1          ATCGGCTTTATTGCTCAAAGAGACATGGGT-GGAAGAGATGAAGGTTACGATTGGTTGAT
39_20F9ZAF033.ab1          ATCGGCTTTATTGCTCAAAGAGACATGGGT-GGAAGAGATGAAGGTTACGATTGGTTGAT
39_20F9ZAF032.ab1          ATCGGCTTTATTGCTCAAAGAGACATGGGT-GGAAGAGATGAAGGTTACGATTGGTTGAT
39_20F9ZAF077.ab1          ATCGGCTTTATTGCTCAAAGAGACATGGGT-GGAAGAGATGAAGGTTACGATTGGTTGAT
39_20F9ZAF075.ab1          ATCGGCTTTATTGCTCAAAGAGACATGGGT-GGAAGAGATGAAGGTTACGATTGGTTGAT
39_20F9ZAF038.ab1          ATCGGCTTTATTGCTCAAAGAGACATGGGT-GGAAGAGATGAAGGTTACGATTGGTTGAT
LBYR36_20F9ZAF068.ab1      ATCGGCTTTATTGCTCAAAGAGACATGGGT-GGAAGAGATGAAGGTTACGATTGGTTGAT

36_20F9ZAF028.ab1          TATGACACCCGGTGTGGGTTTAGATGACAAGGGAGACGCATTGGGTCAACAGTATAGAAC
36_20F9ZAF024.ab1          TATGACACCCGGTGTGGGTTTAGATGACAAGGGAGACGCATTGGGTCAACAGTATAGAAC
36_20F9ZAF023.ab1          TATGACACCCGGTGTGGGTTTAGATGACAAGGGAGACGCATTGGGTCAACAGTATAGAAC
36_20F9ZAF069.ab1          TATGACACCCGGTGTGGGTTTAGATGACAAGGGAGACGCATTGGGTCAACAGTATAGAAC
36_20F9ZAF071.ab1          TATGACACCCGGTGTGGGTTTAGATGACAAGGGAGACGCATTGGGTCAACAGTATAGAAC
URA3                       TATGACACCCGGTGTGGGTTTAGATGACAAGGGAGACGCATTGGGTCAACAGTATAGAAC
39_20F9ZAF076.ab1          TATGACACCCGGTGTGGGTTTAGATGACAAGGGAGACGCATTGGGTCAACAGTATAGAAC
39_20F9ZAF033.ab1          TATGACACCCGGTGTGGGTTTAGATGACAAGGGAGACGCATTGGGTCAACAGTATAGAAC
39_20F9ZAF032.ab1          TATGACACCCGGTGTGGGTTTAGATGACAAGGGAGACGCATTGGGTCAACAGTATAGAAC
39_20F9ZAF077.ab1          TATGACACCCGGTGTGGGTTTAGATGACAAGGGAGACGCATTGGGTCAACAGTATAGAAC
39_20F9ZAF075.ab1          TATGACACCCGGTGTGGGTTTAGATGACAAGGGAGACGCATTGGGTCAACAGTATAGAAC
39_20F9ZAF038.ab1          TATGACACCCGGTGTGGGTTTAGATGACAAGGGAGACGCATTGGGTCAACAGTATAGAAC
LBYR36_20F9ZAF068.ab1      TATGACACCCGGTGTGGGTTTAGATGACAAGGGAGACGCATTGGGTCAACAGTATAGAAC
```

```
36_20F9ZAF028.ab1        CGTGGATGATGTGGTCTCTACAGGATCTGACATTATTATTGTTGGAAGAGGACTATTTGC
36_20F9ZAF024.ab1        CGTGGATGATGTGGTCTCTACAGGATCTGACATTATTATTGTTGGAAGAGGACTATTTGC
36_20F9ZAF023.ab1        CGTGGATGATGTGGTCTCTACAGGATCTGACATTATTATTGTTGGAAGAGGACTATTTGC
36_20F9ZAF069.ab1        CGTGGATGATGTGGTCTCTACAGGATCTGACATTATTATTGTTGGAAGAGGACTATTTGC
36_20F9ZAF071.ab1        CGTGGATGATGTGGTCTCTACAGGATCTGACATTATTATTGTTGGAAGAGGACTATTTGC
URA3                     CGTGGATGATGTGGTCTCTACAGGATCTGACATTATTATTGTTGGAAGAGGACTATTTGC
39_20F9ZAF076.ab1        CGTGGATGATGTGGTCTCTACAGGATCTGACATTATTATTGTTGGAAGAGGACTATTTGC
39_20F9ZAF033.ab1        CGTGGATGATGTGGTCTCTACAGGATCTGACATTATTATTGTTGGAAGAGGACTATTTGC
39_20F9ZAF032.ab1        CGTGGATGATGTGGTCTCTACAGGATCTGACATTATTATTGTTGGAAGAGGACTATTTGC
39_20F9ZAF077.ab1        CGTGGATGATGTGGTCTCTACAGGATCTGACATTATTATTGTTGGAAGAGGACTATTTGC
39_20F9ZAF075.ab1        CGTGGATGATGTGG--TCTACAGGATCTGACATTATTATTGTTGGAAGAGGACTATTTGC
39_20F9ZAF038.ab1        CGTGGATGATGTGGTCTCTACAGGATCTGACATTATTATTGTTGGAAGAGGACTATTTGC
LBYR36_20F9ZAF068.ab1    CGTGGATGATGTGGTCTCTACAGGATCTGACATTATTATTGTTGGAAGAGGACTATTTGC

36_20F9ZAF028.ab1        AAAGGGAAGGGATGCTAAGGTAGAGGGTGAACGTTACAGAAAAGCAGGCTGGGAAGCATA
36_20F9ZAF024.ab1        AAAGGGAAGGGATGCTAAGGTAGAGGGTGAACGTTACAGAAAAGCA-GCTGGGAAGCATA
36_20F9ZAF023.ab1        AAAGGGAAGGGATGCTAAGGTAGAGGGTGAACGTTACAGAAGAGCAGGCTGGGAAGCATA
36_20F9ZAF069.ab1        AAAGGGAAGGGATGCTAAGGTAGAGGGTGAACGTTACAGAAAAGCAGGCTGGGAAGCATA
36_20F9ZAF071.ab1        AAAGGGAAGGGATGCTAAGGTAGAGGGTGAACGTTACAGAAAAGCAGGCTGGGAAGCATA
URA3                     AAAGGGAAGGGATGCTAAGGTAGAGGGTGAACGTTACAGAAAAGCAGGCTGGGAAGCATA
39_20F9ZAF076.ab1        AAAGGGAAGGGATGCTAAGGTAGAGGGTGAACGTTACAGAAGAGCAGGCTGGGAAGCATA
39_20F9ZAF033.ab1        AAAGGGAAGGGATGCTAAGGTAGAGGGTGAACGTTACAGAAGAGCAGGCTGGGAAGCATA
39_20F9ZAF032.ab1        AAAGGGAAGGGATGCTAAGGTAGAGGGTGAACGTTACAGAAAAGCAGGCTGGGAAGCATA
39_20F9ZAF077.ab1        AAAGGGAAGGGATGCTAAGGTAGAGGGTGAACGTTACAGAAAAGCAGGCTGGGAAGCATA
39_20F9ZAF075.ab1        AAAGGGAAGGGATGCTAAGGTAGAGGGTGAACGTTACAGAAAAGCAGGCTGGGAAGCATA
39_20F9ZAF038.ab1        AAAGGGAAGGGATGCTAAGGTAGAGGGTGAACGTTACAGAAAAGCAGGCTGGGAAGCATA
LBYR36_20F9ZAF068.ab1    AAAGGGAAGGGATGCTAAGGTAGAGGGTGAACGTTACAGAAAAGCAGGCTGGGAAGCATA

36_20F9ZAF028.ab1        TTTGAGAAGAT
36_20F9ZAF024.ab1        TTTGAGAAGAT
36_20F9ZAF023.ab1        TTTGAGAAGAT
36_20F9ZAF069.ab1        TTTGAGAAGAT
36_20F9ZAF071.ab1        TTTGAGAAGAT
URA3                     TTTGAGAAGAT
39_20F9ZAF076.ab1        TTTGAGAAGAT
39_20F9ZAF033.ab1        TTTGAGAAGAT
39_20F9ZAF032.ab1        TTTGAGAAGAT
39_20F9ZAF077.ab1        TTTGAGAAGAT
39_20F9ZAF075.ab1        TTTGAGAAGAT
39_20F9ZAF038.ab1        TTTGAGAAGAT
LBYR36_20F9ZAF068.ab1    TTTGAGAAGAT
```

Figure S5. Expression of VirD5CT is mutagenic in yeast.
Sequences of the *URA3* gene from 5-FOA-resistant mutants obtained after expression of VirD5CT in the yeast strains YTAK001 and YTAK002. Sequences were aligned using the program MUSCLE (https://www.ebi.ac.uk/Tools/msa/muscle/). Sequences of mutants of YTAK001 are indicated by code 36, sequences of mutants of YTAK002 are indicated with code 39. URA3, sequence of the *URA3* gene shown in SGD (www.yeastgenome.org); LBYR36, sequence of untransformed YTAK001. Mutations are highlighted in yellow.

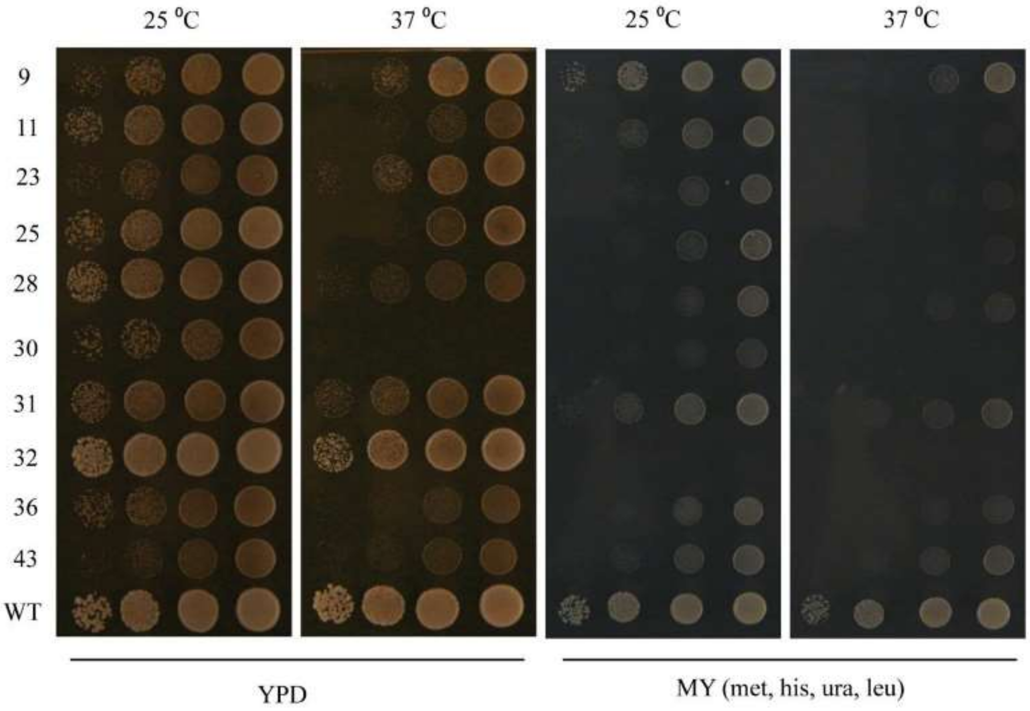

Figure S6. **Growth properties of the mutants found after AMT with the *virD5* containing *Agrobacterium* strain**.
Serial dilutions of the mutants isolated were plated on both rich YPD medium and minimal MY medium and incubated at 25 °C and 37 °C to demonstrate thermosensitive and auxotrophic properties.

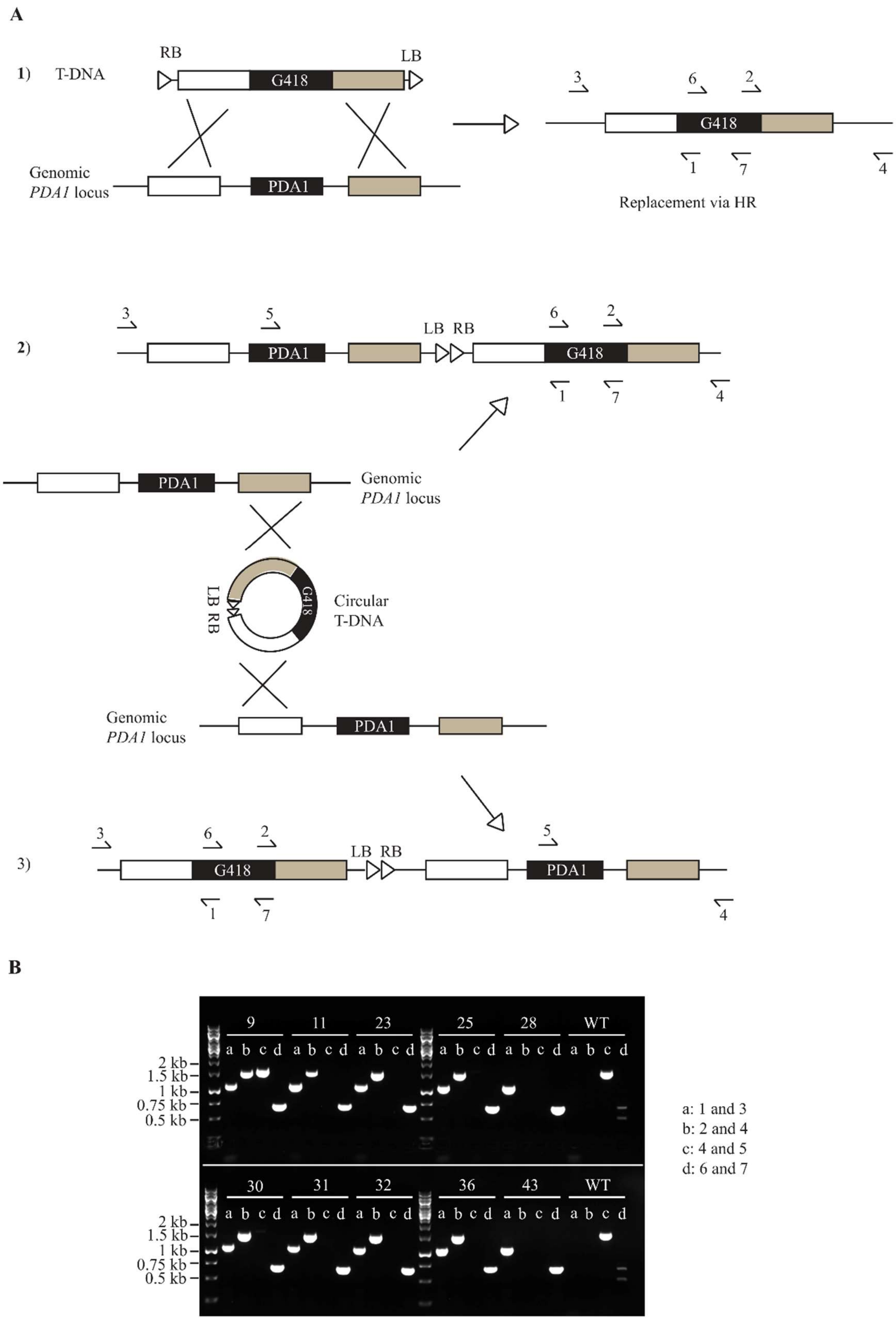
A
1) T-DNA
RB
LB
G418
Genomic
PDA1 locus
PDA1
3
6
2
1
7
4
Replacement via HR
2)
5
LB RB
Circular
T-DNA
3)
B
9
11
23
25
28
WT
30
31
32
36
43
a b c d
2 kb
1.5 kb
1 kb
0.75 kb
0.5 kb
a: 1 and 3
b: 2 and 4
c: 4 and 5
d: 6 and 7

Figure S7. Integration of T-DNA at the *PDA1* locus in chromosome V.

(A) Model depicting the integration of the T-DNA by HR at the *PDA1* locus and (B) PCR analysis confirming integration of the T-DNA at the PDA locus.

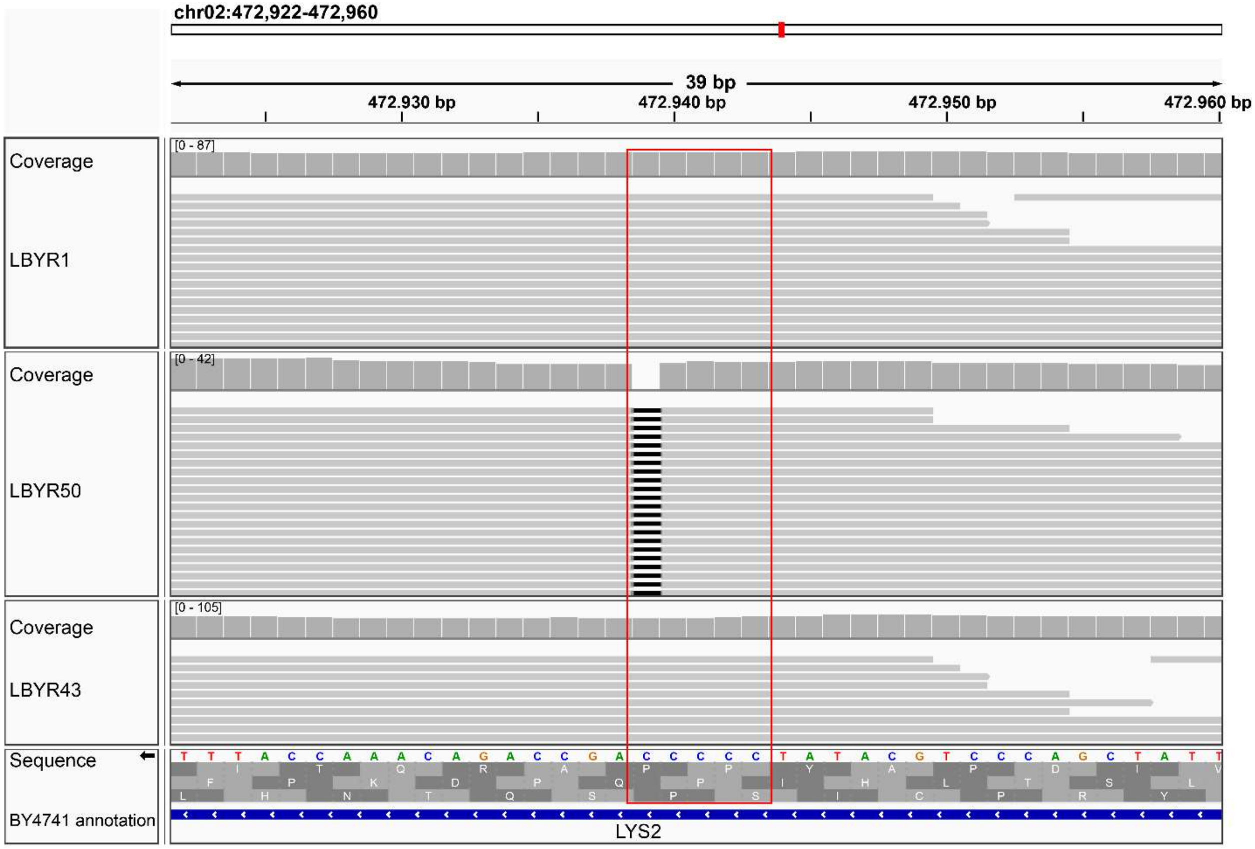

Single nucleotide deletion at 472939 in LBYR50 LYS2

Figure S8. **Genomic sequence of the *LYS2* gene in the auxotrophic yeast mutant LBYR50.** Whole genome analysis of the auxotrophic mutant LBYR50 by Illumina sequencing revealed that the auxotrophy in LBYR50 resides in the *LYS2* gene and is due to a 1 bp deletion from a GGGGG sequence leading to a frameshift in the open reading frame of the *LYS2* gene.

Name	Genotype and description	Source
BY4741	*MATa his3Δ1 leu2Δ0 met15Δ0 ura3Δ0*	(56)
BY4743	*MATa/α* his3Δ1*/his3Δ1 leu2Δ0/leu2Δ0 LYS2/lys2Δ0 met15Δ0/MET15 ura3Δ0/ura3Δ0*	(56)
BY4743::VirD5CT	*MATa/α his3Δ1/his3Δ1 LYS2/lys2Δ0 met15Δ0/MET15 ura3Δ0/ura3Δ0; leu2Δ0/leu2 Δ0::*pRS305-pGAL1-VirD5CT(*LEU2*)	This study
BY4743Δ*rad9*	*MATa/α* his3Δ1*/his3Δ1 leu2Δ0/leu2Δ0 LYS2/lys2Δ0 met15Δ0/MET15 ura3Δ0/ura3Δ0 rad9Δ*::*KanMX/rad9Δ*::*KanMX*	EUROSCARF
W303-1A	*MATa leu2-3,112 trp1-1 can1-100 ura3-1 ade2-1 his3-11,15*	(57)
W303-1A::Rad52-GFP	*MATa leu2-3,112 trp1-1 can1-100 ura3-1 ade2-1 his3-11,15 RAD52-GFP::KanMX*	This study
W303-1A::Rad52-GFP-pGAL1-VirD5CT	*MATa leu2-3,112 trp1-1 can1-100 ura3-1 ade2-1 his3-11,15 RAD52-GFP::KanMX* pMVHis-VirD5CT *(URA3)*	This study
W303-1A::Ddc2-3xGFP	*MATa leu2-3,112 trp1-1 can1-100 ura3-1 ade2-1 his3-11,15 DDC2-3xGFP::KanMX*	This study
W303-1A::Ddc2-3xGFP pGAL1-VirD5CT	*MATa leu2-3,112 trp1-1 can1-100 ura3-1 ade2-1 his3-11,15 DDC2-3xGFP::KanMX* pMVHis-VirD5CT*(URA3)*	This study
YTAK001	*MATa CAN1 his7-2 leu2Δ::KanMX ura3Δ trp1-289 ade2-1 lys2ΔGG2899-2900 agp1*::*URA3-OR1*	(37)
YTAK001 (pRS315-HYG)	*MATa CAN1 his7-2 leu2Δ::KanMX ura3Δ trp1-289 ade2-1* lys2ΔGG2899-2900 *agp1::URA3-OR1* pRS315-HYG(*LEU2*)	This study
YTAK001 (pRS315-HYG-pGAL1-VirD5CT)	*MATa CAN1 his7-2 leu2Δ::KanMX ura3Δ trp1-289 ade2-1 lys2-ΔGG2899-2900 agp1::URA3-OR1* pRS315-HYG-VirD5CT(*LEU2*)	This study

Table S1.
Strains used in this study.

Name	Description	Source
pGPINTAM	Binary vector with a tamoxifen inducible promoter.	(58)
pGPINTAM-VirD5CT	*VirD5CT* (521-833) was amplified using primers VirD5#60 and VirD5#10-2, PCR product digested with NotI was inserted into NotI of pGPINTAMNotI.	This study
pMVHis	High-copy yeast plasmid with a *GAL1* promoter and a *URA3* marker for expression of 6xHis-tagged proteins.	(59)
pMVHis-NLS	SV40NLS fragment was annealed using primers SV40F and SV40R and was digested with BamHI/SalI, the digested fragment was inserted into BamHI/XhoI of pMVHis.	This study
pMVHis-VirD5CT	*VirD5CT* (521-833) was amplified using primers VirD5#56 and VirD5#52, PCR product digested with XmaI and XbaI was inserted into XmaI/XbaI of pMVHis	This study
pMVHis-NES-VirD5CT	NES-VirD5CT was amplified using primers VirD5#86 and VirD5#52, PCR product digested with XmaI and XbaI was inserted into XmaI/XbaI of pMVHis	This study
pMVHis-NES-VirD5CT-NLS	NES-VirD5CT without stop codon was amplified using primers VirD5#86 and VirD5#58, PCR product digested with XmaI and BamHI was inserted into XmaI/BamHI of pMVHis-NLS	This study
pRS305	Yeast integrative plasmid with a *LEU2* marker.	(60)
pRS305-pGAL1-VirD5CT	*pGAL1-His-VirD5CT-Ter* cassette was amplified using primers pMVHisVirD5FW and pMVHisVirD5REV and using pMVHis-VirD5CT as template, PCR product digested with SpeI and SalI was inserted into SpeI/SalI of pRS305.	This study
pRS306	Yeast integrative plasmid with a *URA3* marker.	(60)
pRS315-pGAL1-VirD5CT	*pGAL1-His-VirD5CT-Ter* cassette was amplified using primers pMVHisVirD5FW and pMVHisVirD5REV and using pMVHis-VirD5CT as template, PCR product digested with SpeI and SalI was inserted into SpeI/SalI of pRS315.	This study
pRS306-pGAL1-VirD5CT	*pGAL1-His-VirD5CT-Ter* cassette was amplified using primers pMVHisVirD5FW and pMVHisVirD5REV and	This study

Table S2.
Plasmids used in this study.

Name	Sequence (5'-3')
VirD5#10-2	AAAGCGGCCGCTCAGCGTTTAAACGC
VirD5#23-2	ACGCGTCGACTCAGCGTTTAAAC
VirD5#52	TGCTCTAGATTAGCGTTTAAACGCTTTGTC
VirD5#56	CCGCCCGGGGACCGTTACAGCTTCTATCC
VirD5#58	CGCGGATCCGCGTTTAAACGCTTTGTC
VirD5#60	AAAGCGGCCGCAAACCGTTACAGCTTCTATCC
VirD5#74	GGACTAGTACCGTTACAGCTTCTATCC
VirD5#85	CCATCGATCTACAGCTGCCACCGCTAGAGAGACTTAC GTTAGGAGCAACCGTTACAGCTTCTATCC
VirD5#86	CCGCCCGGGGCTACAGCTGCCACCGCTAGAGAGACTT ACGTTAGGAGCAACCGTTACAGCTTCTATCC
pMVHisVirD5FW	GGACTAGTTCACGCTGGGCGTAACCACCA
pMVHisVirD5REV	ACGCGTCGACATTAAAGCCTTCGAGCGTCCC
SV40F	CGCGGATCCCCAAAAAAGAAGAGAAAGGTCGTTGTT AAATAGGTCGACGCGT
SV40R	ACGCGTCGACCTATTTAACAACGACCTTTCTCTTCTTT TTTGGGGATCCGCG
Pri1FW	CCGCCCGGGCCTTGCTGGTTCAACCATTAC
Pri1REV	CCGCCCGGGACCACGGCACCATTTTACAC
Pol1FW	CCGCCCGGGAGTTCTGAGTAGCGGTGAAAC
Pol1REV	CCGCCCGGGAGTGCTTCACAAGATTTAAG

Primers 1-7 for analysis of the *PDA1* locus have been described previously (38).

Table S3.
Primers used in this study.

REFERENCES AND NOTES

1. M. W. Bevan, M.-D. Chilton, T-DNA of the agrobacterium Ti and Ri plasmids. *Annu. Rev. Genet.* **16**, 357–384 (1982).

2. E. W. Nester, T. Kosuge, Plasmids specifying plant hyperplasias. *Annu. Rev. Microbiol.* **35**, 531–565 (1981).

3. T. Tzfira, Y. Rhee, M. H. Chen, T. Kunik, C. Citovsky, Nucleic acid transport in plant-microbe interactions: The molecules that walk through the walls. *Annu. Rev. Microbiol.* **54**, 187–219 (2000).

4. S. B. Gelvin, Plant proteins involved in *Agrobacterium*-mediated genetic transformation. *Annu. Rev. Phytopathol.* **48**, 45–68 (2010).

5. C. A. McCullen, A. N. Binns, *Agrobacterium tumefaciens* and plant cell interactions and activities required for interkingdom macromolecular transfer. *Annu. Rev. Cell Dev. Biol.* **22**, 101–127 (2006).

6. P. J. J. Hooykaas, R. A. Schilperoort, Agrobacterium and plant genetic engineering. *Plant Mol. Biol.* **19**, 15–38 (1992).

7. P. Bundock, A. den Dulk-Ras, A. Beijersbergen, P. J. J. Hooykaas, Trans-kingdom T-DNA transfer from *Agrobacterium tumefaciens* to *Saccharomyces cerevisiae*. *EMBO J.* **14**, 3206–3214 (1995).

8. M. J. de Groot, P. Bundock, P. J. J. Hooykaas, A. G. Beijersbergen, *Agrobacterium tumefaciens*-mediated transformation of filamentous fungi. *Nat. Biotechnol.* **16**, 839–842 (1998).

9. A. Idnurm, A. M. Bailey, T. C. Cairns, C. E. Elliott, G. D. Foster, G. Ianiri, J. Jeon, A silver bullet in a golden age of functional genomics: The impact of *Agrobacterium*-mediated transformation of fungi. *Fungal. Biol. Biotechnol.* **4**, 6 (2017).

10. R. Mayerhofer, Z. Koncz-Kalman, C. Nawrath, G. Bakkeren, A. Crameri, K. Angelis, G. P. Redei, J. Schell, B. Hohn, C. Koncz, T-DNA integration: A mode of illegitimate recombination in plants. *EMBO J.* **10**, 697–704 (1991).

11. P. J. Krysan, J. C. Young, M. R. Sussman, T-DNA as an insertional mutagen in Arabidopsis. *Plant Cell*, **11**, 2283–2290 (1999).

12. A. Idnurm, F. J. Walton, A. Floyd, J. L. Reedy, J. Heitman, Identification of *ENA1* as a virulence gene of the human pathogenic fungus *Cryptococcus neoformans* through signature-tagged insertional mutagenesis. *Eukaryot. Cell* **8**, 315–326 (2009).

13. L. Márton, M. Hrouda, A. Pécsváradi, M. Czakó, T-DNA-insert-independent mutations induced in transformed plant cells during Agrobacterium co-cultivation. *Transgenic Res.* **3**, 317–325 (1994).

14. H. J. Schouten, H. vande Geest, S. Papadimitriou, M. Bemer, J. G. Schaart, M. J. Smulders, G. S. Perez, E. Schijlen, Re-sequencing transgenic plants revealed rearrangements at T-DNA inserts, and integration of a short T-DNA fragment, but no increase of small mutations elsewhere. *Plant Cell Rep.* **36**, 493–504 (2017).

15. N. Jiang, Y. S. Lee, E. Mukundi, F. Gomez-Cano, L. Rivero, E. Grotewold, Diversity of genetic lesions characterizes new *Arabidopsis* flavonoid pigment mutant alleles from T-DNA collections. *Plant Sci.* **291**, 110335 (2020).

16. F. J. Wei, L.Y. Kuang, H. M. Oung, S. Y. Cheng, H. P. Wu, L. T. Huang, Y. T. Tseng, W. Y. Chiou, V. Hsieh-Feng, C. H. Chung, S. M. Yu, L.Y. Lee, S. B. Gelvin, Y. I. Hsing, Somaclonal variation does not preclude the use of rice transformants for genetic screening. *Plant J.* **85**, 648–659 (2016).

17. S. Imai, T. Ooki, N. Murata-Kamiya, D. Komura, K. Tahmina, W. Wu, A. Takahashi-Kanemitsu, C.T. Knight, A. Kunita, N. Suzuki, A. A. Del Valle, M. Tsuboi, M. Hata, Y. Hayakawa, N. Ohnishi, K. Ueda, M. Fukayama, T. Ushiku, S. Ishikawa, M. Hatakeyama,

Helicobacter pylori CagA elicits BRCAness to induce genome instability that may underlie bacterial gastric carcinogenesis. *Cell Host Microbe* **29**, 941–958.e10 (2021).

18. B. Schrammeijer, A. Beijersbergen, K. B. Idler, L. S. Melchers, D. V. Thompson, P. J. J. Hooykaas, Sequence analysis of the *vir*-region from *Agrobacterium tumefaciens* octopine Ti plasmid pTi15955. *J. Exp. Bot.* **51**, 1167–1169 (2000).

19. A. C. Vergunst, B. Schrammeijer, A. den Dulk-Ras, C. M. de Vlaam, T. J. Regensburg-Tuïnk, P. J. J. Hooykaas, VirB/D4-dependent protein translocation from *Agrobacterium* into plant cells. *Science* **290**, 979–982 (2000).

20. A. C. Vergunst, M. C. van Lier, A. den Dulk-Ras, T.A. Stüve, A. Ouwehand, P. J. J. Hooykaas, Positive charge is an important feature of the C-terminal transport signal of the VirB/D4-translocated proteins of Agrobacterium. *Proc. Natl. Acad. Sci. U.S.A.* **102**, 832–837 (2005).

21. M. R. Roushan, M. A. M. de Zeeuw, P. J. J. Hooykaas, G. P. H. van Heusden, Application of phiLOV2.1 as a fluorescent marker for visualization of *Agrobacterium* effector protein translocation. *Plant J.* **96**, 685–699 (2018).

22. X. Zhang, "Functional analysis of *Agrobacterium tumefaciens* virulence protein VirD5," thesis, Leiden University, Leiden, The Netherlands (2016).

23. X. Zhang, G. P. H. van Heusden, P. J. J. Hooykaas, Virulence protein VirD5 of *Agrobacterium tumefaciens* binds to kinetochores in host cells via an interaction with Spt4. *Proc. Natl. Acad. Sci. U.S.A.* **114**, 10238–10243 (2017).

24. Y. Wang, W. Peng, X. Zhou, F. Huang, L. Shao, M. Luo, The putative *Agrobacterium* transcriptional activator-like virulence protein VirD5 may target T-complex to prevent the degradation of coat proteins in the plant cell nucleus. *New Phytol.* **203**, 1266–1281 (2014).

25. S. Magori, V. Citovsky, Agrobacterium counteracts host-induced degradation of its effector F-box protein. *Sci. Signal.* **4**, ra69 (2011).

26. Y. Wang, S. Zhang, F. Huang, X. Zhou, Z. Chen, W. Peng, M. Luo, VirD5 is required for efficient *Agrobacterium* infection and interacts with Arabidopsis VIP2. *New Phytol.* **217**, 726–738 (2018).

27. L. B. Crotti, M. A. Basrai, Functional roles for evolutionarily conserved Spt4p at centromeres and heterochromatin in *Saccharomyces cerevisiae*. *EMBO J.* **23**, 1804–1814 (2004).

28. X. Zhang, P. J. J. Hooykaas, The agrobacterium VirD5 protein hyperactivates the mitotic Aurora kinase in host cells. *New Phytol.* **222**, 1551–1560 (2019).

29. M. Lisby, R. Rothstein, U. H. Mortensen, Rad52 forms DNA repair and recombination centers during S phase. *Proc. Natl. Acad. Sci. U.S.A.* **98**, 8276–8282 (2001).

30. L. Letavayová, E. Marková, K. Hermanská, V. Vlcková, D. Vlasáková, M. Chovanec, J. Brozmanová, Relative contribution of homologous recombination and non-homologous end-joining to DNA double-strand break repair after oxidative stress in *Saccharomyces cerevisiae*. *DNA Repair* **5**, 602–610 (2006).

31. G.M. Jones, J. Stalker, S. Humphray, A. West, T. Cox, J. Rogers, I. Dunham, G. Prelich, A systematic library for comprehensive overexpression screens in *Saccharomyces cerevisiae*. *Nat. Methods* **5**, 239–241 (2008).

32. M. O'Donnell, H. Li, The eukaryotic replisome goes under the microscope. *Curr. Biol.* **26**, R247–R256 (2016).

33. V. Bianchi, E. Pontis, P. Reichard, Changes of deoxyribonucleoside triphosphate pools induced by hydroxyurea and their relation to DNA synthesis. *J. Biol. Chem.* **261**, 16037–16042 (1986).

34. H. Biswas, G. Goto, W. Wang, P. Sung, K. Sugimoto, Ddc2ATRIP promotes Mec1ATR activation at RPA-ssDNA tracts. *PLOS Genet.* **15**, e1008294 (2019).

35. A. Ait Saada, S. Lambert, A. M. Carr, Preserving replication fork integrity and competence via the homologous recombination pathway. *DNA Repair* **71**, 135–147 (2018).

36. R. González-Prieto, A. M. Muñoz-Cabello, M. J. Cabello-Lobato, F. Prado, Rad51 replication fork recruitment is required for DNA damage tolerance. *EMBO J.* **32**, 1307–1321 (2013).

37. S. A. N. McElhinny, D. Kumar, A. B. Clark, D. L. Watt, B. E. Watts, E. B. Lundström, E. Johansson, A. Chabes, T. A. Kunkel, Genome instability due to ribonucleotide incorporation into DNA. *Nat. Chem. Biol.* **6**, 774–781 (2010).

38. R. J. Kokoska, L. Stefanovic, J. DeMai, T. D. Petes, Increased rates of genomic deletions generated by mutations in the yeast gene encoding DNA polymerase delta or by decreases in the cellular levels of DNA polymerase delta. *Mol. Cell. Biol.* **20**, 7490–7504 (2000).

39. H. Van Attikum, P. J. J. Hooykaas, Genetic requirements for the targeted integration of *Agrobacterium* T-DNA in *Saccharomyces cerevisiae*. *Nucl. Acids Res.* **31**, 826–832 (2003).

40. F. J. Lemoine, N. P. Degtyareva, K. Lobachev, T. D. Petes, Chromosomal translocations in yeast induced by low levels of DNA polymerase a model for chromosome fragile sites. *Cell* **120**, 587–598 (2005).

41. W. Song, M. Dominska, P. W. Greenwell, T. D. Petes, Genome-wide high-resolution mapping of chromosome fragile sites in *Saccharomyces cerevisiae*. *Proc. Natl. Acad. Sci. U.S.A.* **111**, E2210–E2218 (2014).

42. D. Q. Zheng, K. Zhang, X. C. Wu, P. A. Mieczkowski, T. D. Petes, Global analysis of genomic instability caused by DNA replication stress in *Saccharomyces cerevisiae*. *Proc. Natl. Acad. Sci. U.S.A.* **113**, E8114–E8121 (2016).

43. A. Aguilera, The connection between transcription and genomic instability. *EMBO J.* **21**, 195–201 (2002).

44. Y. Hu, B. Lacroix, V. Citovsky, Modulation of plant DNA damage response gene expression during Agrobacterium infection. *Biochem. Biophys. Res. Commun.* **554**, 7–12 (2021).

45. F. Ramos, L. Durán, M. Sánchez, A. Campos, D. Hernández-Villamor, F. Antequera, A. Clemente-Blanco, Genome-wide sequencing analysis of Sgs1, Exo1, Rad51, and Srs2 in DNA repair by homologous recombination. *Cell Rep.* **38**, 110201 (2022).

46. C. S. Gilbert, C. M. Green, N. F. Lowndes, Budding yeast Rad9 is an ATP-dependent Rad53 activating machine. *Mol. Cell* **8**, 129–136 (2001).

47. M. Villa, D. Bonetti, M. Carraro, M. P. Longhese, Rad9/53BP1 protects stalled replication forks from degradation in Mec1/ATR-defective cells. *EMBO Rep.* **19**, 351–367 (2018).

48. I. B. Bentsen, I. Nielsen, M. Lisby, H. B. Nielsen, S. S. Gupta, K. Mundbjerg, A. H. Andersen, L. Bjergbaek, MRX protects fork integrity at protein-DNA barriers, and its absence causes checkpoint activation dependent on chromatin context. *Nucl. Acids Res.* **41**, 3173–3189 (2013).

49. S. Zhang, Z. Chen, F. Huang, Y. Wang, M. Luo, F-Box gene D5RF is regulated by Agrobacterium virulence protein VirD5 and essential for Agrobacterium-mediated plant transformation. *Int. J. Mol. Sci.* **21**, 6731 (2020).

50. X. Tang, G. Liu, J. Zhou, Q. Ren, Q. You, L. Tian, X. Xin, Z. Zhong, B. Liu, X. Zheng, D. Zhang, A. Malzahn, Z. Gong, Y. Qi, T. Zhang, Y. Zhang, A large-scale whole-genome sequencing analysis reveals highly specific genome editing by both Cas9 and Cpf1 (Cas12a) nucleases in rice. *Genome Biol.* **19**, 84 (2018).

51. G. I. Lang, A. W. Murray, Estimating the per-base-pair mutation rate in the yeast Saccharomyces cerevisiae. *Genetics* **178**, 67–82 (2008).

52. M. R. Roushan, S. Shao, I. Poledri, P. J. J. Hooykaas, G. P. H. van Heusden, Increased *Agrobacterium*-mediated transformation of *Saccharomyces cerevisiae* after deletion of the yeast *ADA2* gene. *Lett. Appl. Microbiol.* **74**, 228–237 (2022).

53. G. R. Lazo, P. A. Stein, R. A. Ludwig, A DNA transformation–competent *Arabidopsis* genomic library in *Agrobacterium*. *Bio/technology* **9**, 963–967 (1991).

54. H. Li, Aligning sequence reads, clone sequences and assembly contigs with BWA-MEM. arXiv:1303.3997 [q-bio.GN] (2013).

55. E. Garrison, G. Marth, Haplotype-based variant detection from short-read sequencing. arXiv:1207.3907 [q-bio.GN] (2012).

56. C. B. Brachmann, A. Davies, G. J. Cost, E. Caputo, J. Li, P. Hieter, J. D. Boeke, Designer deletion strains derived from *Saccharomyces cerevisiae* S288C: A useful set of strains and plasmids for PCR-mediated gene disruption and other applications. *Yeast* **14**, 115–132 (1998).

57. B.J. Thomas, R. Rothstein, Elevated recombination rates in transcriptionally active DNA. *Cell* **56**, 619–630 (1989).

58. J. Friml, X. Yang, M. Michniewicz, D. Weijers, A. Quint, O. Tietz, R. Benjamins, P. B. Ouwerkerk, K. Ljung, G. Sandberg, P. J. J. Hooykaas, K. Palme, R. Offringa, A PINOID-dependent binary switch in apical-basal PIN polar targeting directs auxin efflux. *Science* **306**, 862–865 (2004).

59. M. J. van Hemert, A. M. Deelder, C. Moolenaar, H. Y. Steensma, G. P. H. van Heusden, Self-association of the spindle pole body-related intermediate filament protein Fin1p and its phosphorylation-dependent interaction with 14-3-3 proteins in yeast. *J. Biol. Chem.* **278**, 15049–15055 (2003).

60. R. S. Sikorski, P. Hieter, A system of shuttle vectors and yeast host strains designed for efficient manipulation of DNA in *Saccharomyces cerevisiae*. *Genetics* **122**, 19–27 (1989).
